# Reporting of nutritional screening, status, and intake in trials of nutritional and physical rehabilitation following critical illness: a systematic review

**DOI:** 10.1016/j.ajcnut.2024.12.028

**Published:** 2024-12-31

**Authors:** Reema Rabheru, Anne Langan, Judith Merriweather, Bronwen Connolly, Kevin Whelan, Danielle E Bear

**Affiliations:** 1 Department of Nutrition and Dietetics, Guy’s and St Thomas’ NHS Foundation Trust, London, United Kingdom; 2 Department of Nutritional Sciences, King’s College London, London, United Kingdom; 3 Department of Nutrition and Dietetics, Barts Health NHS Trust, London, United Kingdom; 4 Critical Care, Royal Infirmary of Edinburgh, Edinburgh, United Kingdom; 5 Department of Nutrition and Dietetics, Royal Infirmary of Edinburgh, Edinburgh, United Kingdom; 6 Wellcome-Wolfson Institute for Experimental Medicine, School of Medicine, Dentistry and Biomedical Sciences, Queen’s University Belfast, Belfast, United Kingdom; 7 Department of Physiotherapy, The University of Melbourne, Australia; 8 Department of Critical Care, Guy’s and St Thomas’ NHS Foundation Trust, London, United Kingdom

**Keywords:** critical illness, nutritional intervention, physical rehabilitation intervention, ICU, physical recovery, functional recovery

## Abstract

**Background:**

Surviving critical illness leads to prolonged physical and functional recovery with both nutritional and physical rehabilitation interventions for prevention and treatment being investigated. Nutritional status and adequacy may influence outcome, but no consensus on which nutritional-related variables should be measured and reported in clinical trials exists.

**Objectives:**

This study aimed to undertake a systematic review investigating the reporting of nutritional screening, nutritional status, and nutritional intake/delivery in randomized controlled trials (RCTs) evaluating nutritional and/or physical rehabilitation on physical and functional recovery during and following critical illness.

**Methods:**

Five electronic databases (MEDLINE, Web of Science, EMBASE, CINAHL, and Cochrane) were searched (last update 9 August, 2023). Search terms included both free text and standardized indexed terms. Studies included were RCTs assessing nutritional and/or physical interventions either during or following intensive care unit (ICU) admission in adults (18 y or older) with critical illness, and who required invasive mechanical ventilation for any duration during ICU admission. Study quality was assessed using the Cochrane Collaboration Risk of Bias tool for RCTs and descriptive data synthesis was performed and presented as counts (%). n t

**Results:**

In total, 123 RCTs (30 nutritional, 87 physical function, and 6 combined) were included. Further, ≥1 nutritional variable was measured and/or reported in 99 (80%) of the studies including BMI (*n* = 69), body weight (*n* = 57), nutritional status (*n* = 11), nutritional risk (*n* = 10), energy delivery (*n* = 41), protein delivery (*n* = 35), handgrip strength (*n* = 40), and other nutritional-related muscle variables (*n* = 41). Only 3 studies were considered to have low risk of bias in all categories.

**Conclusions:**

Few RCTs of physical rehabilitation measure and report nutritional or related variables. Future studies should measure and report specific nutritional factors that could impact physical and functional recovery to support interpretation where studies do not show benefit.

This protocol was preregistered at PROSPERO as CRD42022315122.

## Introduction

Evolution in the management of critical illness has resulted in a reduction in intensive care unit (ICU) mortality [1]. However, the increasing number of patients surviving critical illness has led to prolonged recovery with an increase in physical and functional disability and reduced quality of life that can persist for ≤10 y following an ICU admission [[1], [2], [3]]. The etiology of impaired recovery in ICU survivors is multifactorial and includes muscle wasting and ICU-acquired weakness [4]. Both nutritional and physical rehabilitation during and following critical illness have the potential to improve physical, functional, and quality of life recovery following critical illness [5].

Current evidence investigating nutritional and physical rehabilitation is contradictory. Outcomes of studies of nutritional interventions vary depending upon the biological plausibility and mechanism of the intervention and its dose, timing, route and duration [6]. Outcomes of studies of physical rehabilitation are also highly dependent on the dose and intensity of the intervention and the definition of “usual care” in the comparator group. For both nutritional and physical rehabilitation, patient-related factors also impact the outcome of studies, including the nutritional, physical, and functional status of the patient preadmission, the patient’s ability to perform the nutritional or physical intervention including motivation, beliefs regarding the benefits and risks, and environmental factors [7].

For both nutritional and physical rehabilitation interventions, it is likely that nutritional-related factors such as nutritional status, body composition, and nutritional intake or delivery will influence physical and functional recovery. Plausibly, these interventions may be more effective in patients who are well nourished, have greater muscle mass, and receive adequate energy and protein provision. However, there is no consensus on which specific nutritional-related factors might impact response to nutritional and physical rehabilitation interventions and, therefore, what factors should be measured, reported, or considered when investigating interventions to improve physical and functional recovery in critically ill patients.

Therefore, the aim was to undertake a systematic review to investigate the reporting of nutritional screening, nutritional status, and nutritional intake/delivery in randomized controlled trials (RCTs) evaluating nutritional and/or physical rehabilitation interventions on physical and physical functional recovery during and following critical illness.

## Methods

A systematic review was performed according to the Cochrane Handbook for Systematic Reviews of Interventions [8] and reported in line with the PRISMA guidelines [9] (Supplemental Appendixes I and II), A protocol was developed in discussion with the research group and preregistered at PROSPERO (CRD42022315122). Owing to differences in search terms, eligibility and nature of the interventions, the searches, study selection, and data extraction for the nutritional and physical rehabilitation studies were conducted separately, but followed the same process.

### Search strategy

A literature search was undertaken combining the 4 main concepts: *1*) population: adult critically ill patients, *2*) interventions: nutritional and/or physical rehabilitation, *3*) outcomes: physical or physical functional recovery, and *4*) study designs: RCTs. Synonyms and related phrases using both British and American spellings were used. Search terms included both free text and standardized indexed terms (e.g. MeSH). The final search strategies were reviewed by a librarian at King’s College London and are available in Supplemental Appendix III. Five electronic databases (MEDLINE, Web of Science, EMBASE, CINAHL, and Cochrane) were searched from inception until 9 August, 2023. No date range restrictions were applied.

Two international trial databases (Clinical Trials and International Clinical Trials Registry Platform) were also searched for completed, unpublished studies. Reference lists of relevant studies and previous systematic reviews were manually reviewed for additional studies not identified in the electronic search.

### Eligibility criteria

Studies were eligible if they were RCTs investigating interventions of nutritional or physical rehabilitation (or both) either during or following ICU to investigate the impact on physical or physical functional (herewith referred to as function/functional throughout) recovery in adults. The eligibility criteria are summarized in Table 1.

**TABLE 1:** Inclusion and exclusion criteria.

Characteristic	Inclusion criteria	Exclusion criteria
Population	Adults (≥18 y) admitted to ICU with critical illness, and who required invasive mechanic ventilation for any duration during the ICU admission	Child or adolescent populations Animal studies
Intervention	Studies were included where the intervention aimed to promote physical or functional recovery during and after critical illness and was commenced either during or following ICU admission, but the duration of intervention delivery may be of any timeframe. Nutritional interventions included any that related to the timing, type, composition, route of nutritional support (e.g. enteral, parenteral, or oral), or other intervention in any form or any combination. Physical rehabilitation interventions to promote physical or functional recovery during and after critical illness. Examples include (but not limited to): early mobilization including passive or active exercises, progression of functional mobility, strengthening exercise, cycling, electrical muscle stimulation, group exercise, hydrotherapy, use of interactive technology	Studies investigating interventions of respiratory muscle training
Comparator	The comparator included standard care, placebo, sham, or other comparable interventions	Nil
Outcome	Studies measuring physical or physical functional recovery, defined as “the impact of disease/condition on physical activities of daily living.” Physical recovery included the impact on muscle strength or architecture, and functional recovery included (but not limited to) ability to walk, independence, self-care, performance status, disability index, measures relating to sleep, motor skills, sexual dysfunction, health behavior and management	Nil
Study design	All parallel design RCTs where physical and functional outcomes were reported	Studies reporting only feasibility outcomes Non-RCTs and descriptive commentary (reviews, editorials, and narratives) Conference abstracts, as they would have insufficient content to meet the aim of the review

Abbreviation: ICU, intensive care unit.

### Study selection

Search outcomes from the electronic databases, as well as from backward citation searching, were imported into Covidence (Veritas Health Innovation, Melbourne, Australia). Duplicates were automatically removed before further manual deduplication by 1 investigator (RR).

Titles and abstracts were screened for eligibility independently and in duplicate by 1 researcher (RR) and by 1 member of the review panel (AL, JM, DEB). Potential eligible studies underwent full-text screening independently and in duplicate by the same 2 researchers who undertook title and abstract screening. Any discrepancies were resolved by discussion with a third investigator (DEB, KW).

### Data extraction

Data extraction was performed in duplicate and independently by 1 researcher (RR) and 1 member of the review panel (AL, JM, DEB) using a predeveloped and piloted data collection form. Authors were not contacted for missing or additional data as the aim was to extract whether nutritional data were reported. Data were crosschecked between data extractors for discrepancies, and disagreements were resolved and corrected through discussion.

Data extracted included author details, year of publication, participant characteristics, details of intervention and comparator, and number recruited and analyzed. The major data extraction related to the measurement and reporting of nutritional measures at baseline, end of the intervention, and end of the follow-up period (or changes in these values over these time points), as follows:

• Anthropometry (BMI and body weight);
• Nutritional screening or nutritional risk undertaking using a screening tool (e.g. Malnutrition Universal Screening Tool);
• Nutritional status, either a marker (e.g. body weight, BMI, and weight loss) or the use of an integrative score (e.g. Subjective Global Assessment [SGA] and Global Leadership Initiative on Malnutrition [GLIM]) or other criteria;
• Nutritional intake (e.g. energy and protein delivery or intake);
• Nutritional-related muscle variables (e.g. measures of muscle mass and muscle strength).

A nutritional variable was considered to have been measured if it was reported as being planned in the study methods and reported if it was reported within the results section of the published article or in any associated supplemental appendix. Whether these data were used in adjustment of data analysis was also extracted.

### Assessment of risk of bias

Risk of bias was assessed independently and in duplicate by 1 researcher (RR) and 1 member of the review panel (AL, JM, DEB) using the Cochrane Collaboration risk-of-bias tool for RCTs [8]. Each study was categorized as low, unclear, or high for the following domains: random sequence generation, allocation concealment, blinding of participants and personnel, blinding of outcome assessment, incomplete outcome data, selective reporting, and other sources of bias. Discrepancies were resolved by consensus between the researchers.

### Data synthesis

The characteristics of the RCTs, including the population, intervention, comparator, outcomes, setting, and any outcomes relevant to the aim of the review (e.g. nutritional screening tool, nutritional status, anthropometry, energy and protein delivery/intake, and nutritional-related variables) were reported descriptively and displayed in a study characteristics table. Meta-analysis was not performed as this systematic review investigated the measurement and reporting of nutritional data, not effectiveness end points. Descriptive data are grouped according to the intervention (e.g. nutritional intervention, physical rehabilitation, and combined intervention). Descriptive comparison of studies of physical interventions undertaken in ICU and post-ICU (i.e. post-ICU discharge in hospital or posthospital discharge) was performed to explore possible differences in study populations. There were no nutritional intervention studies undertaken outside of the ICU, and therefore, such a descriptive comparison of subgroups based upon location was not performed for nutritional interventions. Data are presented as counts (%).

## Results

### Study selection

**FIGURE 1:**
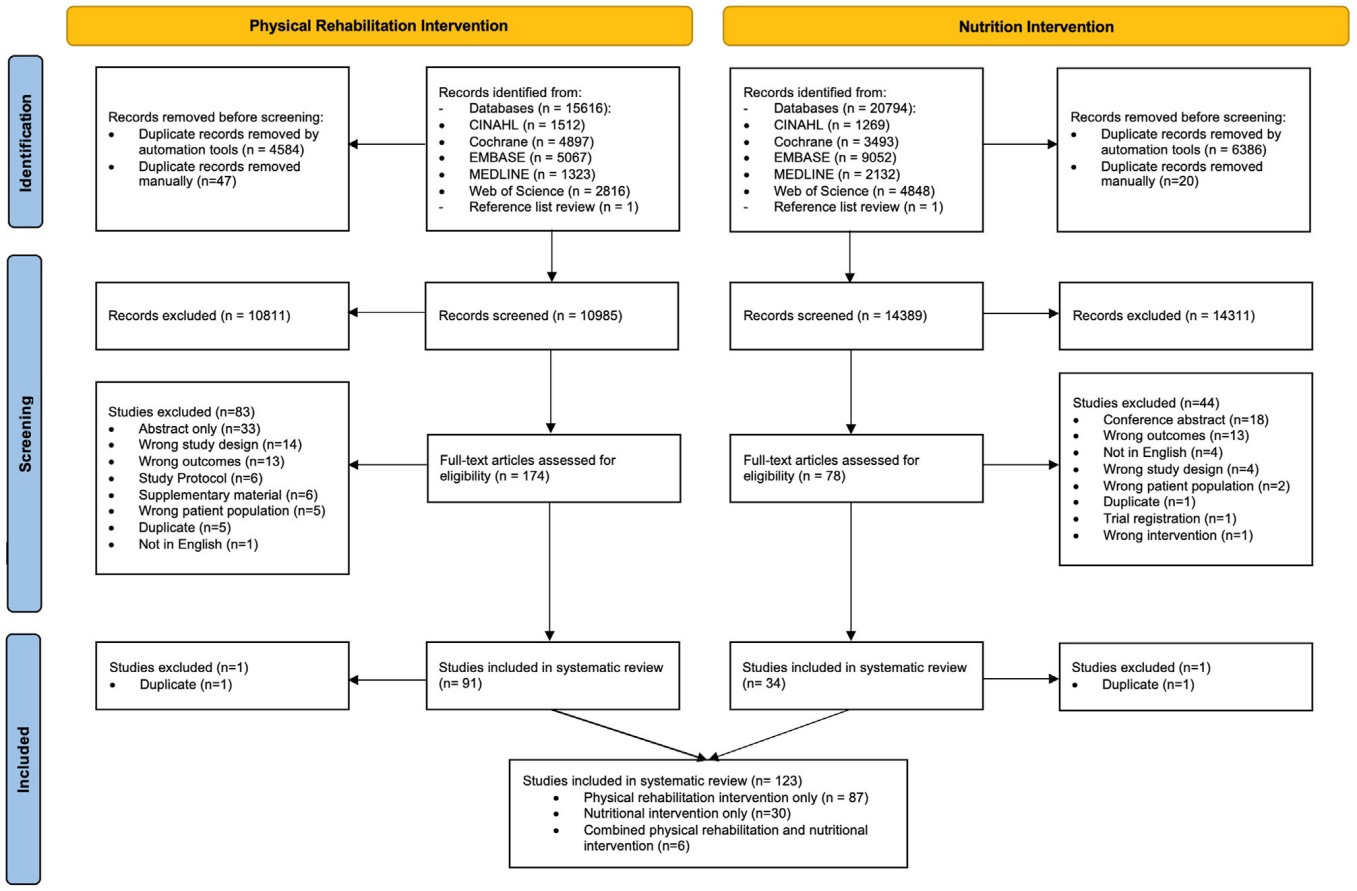
PRISMA flow diagram of study selection process.

For studies of nutritional interventions, 20,794 studies were generated from the search strategy and 1 from back-searching reference lists (Figure 1). After duplicates were removed, 14,389 records were available for title and abstract screening. Of these, 78 records were retrieved for full-text screening, with 34 RCTs being eligible for inclusion.

For studies of physical rehabilitation interventions, 15,616 studies were generated from the search and 1 from back-searching reference lists (Figure 1). After duplicates were removed, 10,985 records were available for title and abstract screening. Of these, 174 records were retrieved for full-text screening, with 91 RCTs being eligible for inclusion.

The eligible studies from both searches were then combined to form 124 eligible studies, of which 1 was excluded due to duplication. Six of the 123 studies were combined physical rehabilitation and nutritional interventions.

### Study characteristics

#### Nutritional intervention studies

From the 30 nutritional intervention studies, 17,855 participants were included, with study size ranging from 8 to 4640 participants [[10], [11], [12], [13], [14], [15], [16], [17], [18], [19], [20], [21], [22], [23], [24], [25], [26], [27], [28], [29], [30], [31], [32], [33], [34], [35], [36], [37], [38], [39]]. All studies were performed in ICU (i.e. during an ICU admission), and most included a mixed medical/surgical clinical population (*n* = 20) [[12], [13], [14], [15], [16], [17],^19^,^20^,^22^,^23^,^25^,^26^,[29], [30], [31], [32], [33], [34], [35], [36]] (Table 2). There was considerable variability in duration of intervention ranging from 6 d to 3 mo. Full study characteristics and intervention data are reported in Table 2.

**TABLE 2:** Study characteristics of nutritional intervention studies.

Citation, year	*n*	Population	Age (y)	Intervention	Comparator	Commencement of intervention	Duration of intervention (d)
Allingstrup et al. (2017) [10]	199	Medical and surgical	I: 63 (51–72)^1^ C: 68 (52–75)^1^	Early goal-directed nutrition, nutritional requirements were estimated using indirect calorimetry and 24-h urinary urea aiming at covering 100% of requirements from the first full trial day using EN and PN	Provided 25kcal/kg EN, supplemented by PN if not met by day 7	ICU	ICU admission to a maximum of 90 d I: 7 (5–22)^1^ C: 7 (4–11)^1^
Berger et al. (2019) [11]	23	Medical and surgical	I: 63 (55–73)^1^ C: 67.5 (62.3–75)^1^	On day 3 of admission, the intervention group that were fed <60% of their energy target by EN, continued to SPN to a target validated by indirect calorimetry	Those fed <60% of their energy target by EN, continued EN to a target validated by indirect calorimetry	ICU	Day 3 to day 9 of ICU admission I: 15.3 (10.6–17.4)^1^ C: 9.5 (7.1–24.4)^1^
Casaer et al. (2011) [12]	4640	Mixed	I: 64 (14) C: 64 (15)	Early initiation of PN within 48 h after ICU admission	Late initiation of PN was not initiated before day 8	ICU	ICU admission I: 4 (2–9)^2^ C: 3 (2–7)
Chapple et al. (2022) [13]	80	Mixed	I: 53.5 (17.6) C: 58.2 (14.2)	Energy dense EN (1.5 kcal/kg)	Routine EN (1.0 kcal/kg)	ICU	ICU admission or ≤28 d
de Azevedo et al. (2019) [14]	120	Mixed	I: 65.0 (18.8) C: 67.4 (18.9)	Optimized calorie and high protein nutrition: caloric intake was determined by indirect calorimetry, and protein intake was established at 2.0 to 2.2 g/kg/d	Standard nutritional care: 25 kcal/kg d of calories and 1.4–1.5 g/kg/d	ICU	ICU admission or ≤14 d I: 21 (13–33)^1^ C: 18 (10–35)^1^
Deane et al. (2020) [15]	3957	Mixed	I: 57.5 (16.5) C: 57.7 (16.4)	Energy dense EN (1.5 kcal/kg)	Routine EN (1.0 kcal/kg)	ICU	ICU admission, until EN is ceased or ≤28 d
Doig et al. (2013) [16]	1358	Mixed	I: 68.4 (15.1) C: 68.6 (14.3)	Early PN started at randomization with targets to be achieved on study day 3	Standard care: route, starting rate, metabolic targets, and composition of nutrition based on current practice in their ICU, independent of the study intervention	ICU	ICU admission I: 8.6 (8.2–9.0)^1^ C: 9.3 (8.9–9.7)^1^
Doig et al. (2015) [17]	472	Mixed	I: 63.3 (15.4) C: 62.7 (16.6)	A continuous infusion of a standard mixture of 100 g/L of l-amino acids, a maximum total daily protein intake of 2.0 g/kg/d was achieved	Standard care: route, starting rate, metabolic targets, and composition of nutrition based on current practice in their ICU, independent of the study intervention	ICU	ICU admission I: 11.6 (10.8–12.5)^1^ C: 10.7 (10.0–11.5)^1^
Doig et al. (2015) [18]	331	Refeeding syndrome	I: 59 (16) C: 61 (16)	Restricted caloric management group: nutritional support directed by a study caloric management protocol, which reduced energy intake to 20 kcal/h for ≥2 d	Nutritional support defined pragmatically, consisting of continuing nutritional support as planned before study enrolment	ICU	ICU admission I: 11.4 (10.5–12.4)^1^ C: 10.0 (9.2–10.9)^1^
Dresen et al. (2021) [19]	42	Mixed	I: 66 (16) C: 64 (15)	Nutrient supply via EN or PN was calculated based on the individual energy expenditure measured by indirect calorimetry and target protein content of 1.8 g protein/kg BW/d	Standard care of 1.2 g protein/kg BW/d	ICU	28 d
Dresen et al. (2022) [20]	42	Mixed	I: 66 (16) C: 64 (15)	Secondary analysis of Dresen et al., 2021 [19] evaluating the potential associations of amino acid patterns. Intervention as per original study	Standard care of 1.2 g protein/kg BW/d	ICU	28 d
Ferrie et al. (2016) [21]	119	Medical and surgical	I: 67.0 (55.5–74.3)^1^ C: 64.5 (49.3–70.0)^1^	24 kcal/kg with a daily amino acid delivery of 1.2 g/kg	26 kcal/kg with a daily amino acid delivery of 0.8 g/kg	ICU	ICU admission or ≤10 d (whichever came first) I: 5.0 (3.0–8.0)^1^ C: 6.0 (3.8–10.0)^1^
Fetterplace et al. (2018) [22]	60	Mixed	I: 55 (13) C: 57 (16)	Volume-based protocol with protein supplementation aiming for 1.5 g/kg/d	Conventional nutritional therapy—hourly rate–based without protein supplementation aiming for 1 g/kg/d	ICU	ICU admission, until EN was no longer indicated or ≤15 d (whichever came first)
Harvey et al. (2014) [23]	2383	Mixed	I: 63.3 (15.1) C: 62.9 (15.4)	PN at 25 kcal/kg	EN at 25 kcal/kg	ICU	ICU admission, ≤5 d or until transition to exclusive oral feeding (whichever came first)
Heming et al. (2022) [24]	35	Sepsis or ARDS	I: 71 (52–83)^1^ C: 71 (62–75)^1^	Mix of 5 amino acids (threonine, cysteine, proline, serine, and leucine) mixed with EN	22 g of maltodextrin as a placebo mixed with EN	ICU	21 d
Hermans et al. (2013) [25]	722	Mixed	I: Group 1: 65 (53–74)^1^ I: Group 2: 62 (45–73)^1^ C: Group 1: 62 (52–73)^1^ C: Group 2: 63 (54–71)^1^	Late initiation of PN. If EN was insufficient by day 7, PN was initiated on day 8 to meet calorie target. Group 1: muscle strength quantified with MRC. Group 2: muscle biopsy was measured	Early initiation of PN. IV glucose 400 kcal day 1, 600 kcal day 2, EN and PN day 3 to meet 100% of calorie target. Group 1: muscle strength quantified with MRC, Group 2: muscle biopsy was measured	ICU	ICU admission or death (whichever came first)
McNelly et al. (2020) [26]	63	Mixed	I: 55.2 (51.0–59.3)^1^ C: 60.3 (56.0–64.1)^1^	Intermittent EN from six 4-hourly feeds per 24 h	Standard continuous EN	ICU	10 d
Nakamura et al. (2020) [27]	50	Medical and surgical	I: 71.8 (12.4) C: 76.6 (12.3)	HMB supplements containing 1.5 g HMB, 7 g arginine, 7 g glutamine twice daily in addition to conventional nutritional therapy plus early rehabilitation with EMS	Conventional nutritional therapy plus early rehabilitation with EMS	ICU	10 d
Nakamura et al. (2021) [28]	117	Medical and surgical	I: 68.3 (14.3) C: 67.9 (14.9)	1.8 g/kg/d protein was targeted	0.9 g/kg/d protein was targeted	ICU	10 d
Needham et al. (2013) [29]	951	Mixed	I: 52 (16) C: 52 (15)	EN initiated at 20 kcal/h	EN initiated at 25 mL/h and advanced to goal rates (20–30 kcal/d of nonprotein calories and 1.2–1.6 g/kg/d of protein) as soon as possible	ICU	Until extubated or day 6 (whichever came first)
Needham et al. (2013) [30]	174	Mixed	I: 48 (14) C: 47 (14)	EN initiated at 20 kcal/h	EN initiated at 25 mL/h and advanced to goal rates (20–30 kcal/d of nonprotein calories and 1.2–1.6 g/kg/d of protein) as soon as possible	ICU	Until extubated or day 6 (whichever came first)
Reid et al. (2016) [31]	39	Mixed	I: 57 (3.1) C: 54 (3.4)	Energy dense EN—1.5 kcal/mL formula delivered at 1 mL/kg IBW	Routine EN 1 kcal/mL formula delivered at 1 mL/kg IBW	ICU	10 d or until EN ceased
Rice et al. (2012) [32]	1000	Mixed	I: 52 (17) C: 52 (16)	Trophic feeding: EN initiated at 10 mL/h, 10–20 kcal/h, for the first 272 patients who also received ω-3	Full EN: total of 240 mL volume per day	ICU	6 d
Ridley et al. (2018) [33]	99	Mixed	I: 59 (17) C: 60 (17)	Supplemental PN: at a rate based on the percentage of estimated energy requirements received from EN in the 24 hours before randomization. These rates corresponded to either 40% or 80% of the estimated requirement	Nutritional therapy in the usual care group followed clinical practice at the participating ICU	ICU	7 d
Uyar et al. (2023) [34]	49	Mixed	I: 61.72 (21.95) C: 59.79 (23.82)	High protein 2.0 g/kg/d	Standard care 1.2 g/kg/d	ICU	ICU admission
Valizade Hasanloei et al. (2021) [35]	100	Mixed	I: 50.36 (14.41) C: 49.60 (13.52)	EN wheat germ–enriched formula set at 25 kcal/kg/IBW	EN standard formula set at 25 kcal/kg/IBW	ICU	From the first day of admission until weaning from the ventilator
Viana et al. (2021) [36]	30	Mixed	All: 64.94 (58.88–71.00)^1^	HMB supplements containing 1.5 g twice daily in addition to conventional nutritional therapy, EN aiming for 1.2 g/kg/protein	Placebo containing 1.5 g maltodextrin twice daily in addition to conventional nutritional therapy, EN aiming for 1.2 g/kg/protein	ICU	From day 4 for day 10, ≤30 d
Wandrag et al. (2019) [37]	8	Trauma	I: 53 (19–58)^1^ C: 60 (28–99)^1^	5 g of leucine-enriched essential amino acid 5 times per day in addition to standard feed	Standard feed only via ICU protocol	ICU	14 d
Wischmeyer et al. (2017) [39]	125	Medical and surgical	I: 55.8 (19.8) C: 55.1 (16.2)	Supplementary PN, EN, and PN adjusted daily to ensure patients received 100% of calories	Standardized EN only with 1.2 ± 0.2 kcal/mL	ICU	7 d
Wittholz et al. (2023) [38]	50	Trauma	I: 52 (36–56)^1^ C: 49.5 (33–58)^1^	3 g of HMB powder. For patients receiving tube feeding, HMB was dissolved in 150 mL of water. For patients ingesting food, HMB was mixed in 150 mL of orange juice	Isocalorically matched placebo, either 150 mL of water for those receiving tube feeding or 150 mL of orange juice for those ingesting food	ICU	28 d or hospital admission I: 25.0 (19.0-40.0)^1^ C: 22.5 (14.0–32)^1^

Values are mean (SD) unless otherwise specified.

Abbreviations: C, comparator; EAA, essential amino acid; EMS, electrical muscle stimulation; HMB, hydroxymethylbutyrate; ICU, intensive care unit; I, intervention; NMES, neuromuscular electrical stimulation; PEPSE, program of enhanced physiotherapy and structured exercise, SPN, Supplementatry Parenteral Nutrition

1 Median (IQR).

The most common nutritional interventions were high protein/amino acids (*n* = 7) [17,[19], [20], [21], [22],^28^,^34^], single amino acids or their metabolites (*n* = 5) [24,27,[36], [37], [38]], energy-restricted nutrition (*n* = 4) [18,29,30,32], and early parenteral nutritional (*n* = 4) [12,16,23,25] (Table 2).

#### Physical intervention studies

The 88 physical rehabilitation studies involved 8448 participants, with individual studies including between 11 and 1917 participants [[40], [41], [42], [43], [44], [45], [46], [47], [48], [49], [50], [51], [52], [53], [54], [55], [56], [57], [58], [59], [60], [61], [62], [63], [64], [65], [66], [67], [68], [69], [70], [71], [72], [73], [74], [75], [76], [77], [78], [79], [80], [81], [82], [83], [84], [85], [86], [87], [88], [89], [90], [91], [92], [93], [94], [95], [96], [97], [98], [99], [100], [101], [102], [103], [104], [105], [106], [107], [108], [109], [110], [111], [112], [113], [114], [115], [116], [117], [118], [119], [120], [121], [122], [123], [124], [125], [126]]. A total of 70 studies were performed in ICU (i.e. during an ICU admission) with most including a mixed clinical population (*n* = 28) (Table 3) [42,44,51,60,63,64,66,71,74,75,78,81,83,86,87,89,92,94,98,99,102,104,105,108,112,118,121,123]. Nine studies were commenced post-ICU discharge on the ward [55,57,72,84,101,113,117,122,125] and 9 posthospital discharge [45,46,58,65,70,76,77,90,91], with most also including a mixed population (*n* = 10) [45,65,70,72,77,84,90,91,101,122] (Table 3). Full study characteristics and intervention data are reported in Table 3.

**TABLE 3:** Study characteristics of physical rehabilitation studies.

Citation, year	*n*	Population	Age (y)	Intervention	Comparator	Commencement of intervention	Duration of intervention (d)
Abu-Khaber et al., 2013 [40]	80	Unknown	I: 59.07 (5.32) C: 57.57 (6.80)	EMS on the quadriceps muscles of both lower extremities	Standard care	ICU	ICU admission I: 9.01 (8.01) C: 11.97 (8.07)
Akar et al., 2017 [41]	30	COPD and respiratory failure	I: 70.00 (12.28) Group 1: 62.75 (6.80) Group 2: 68.00 (17.77)	Active extremity muscle exercise training and NMES	Group 1: NMES alone Group 2: active extremity exercise training alone	ICU	5 d/wk for 20 sessions
Amundadottir et al., 2021 [42]	49	Mixed	I: 62 (50–70)^a^ C: 64 (58–74)^1^	Twice daily upright mobilization including active-assisted and active exercises, and functional, strength, balance, and transfer training	Once daily upright mobilization that started on day 5 after initiation of mechanical ventilation	ICU	ICU admission I: 12.4 (8.4–19.6)^1^ C: 11.0 (7.3–22.8)^1^
Angelopoulos et al., 2013 [43]	31	Systemic inflammatory response syndrome or sepsis	I: 57 (12) C: 63 (12)	High frequency NMES	Medium frequency NMES	ICU	A single 30-min session
Bao et al., 2022 [44]	60	Mixed	Group 1: 52.80 (10.79) Group 2: 51.10 (17.61) C: 52.50 (12.51)	Group 1: received APAT on the lower limbs + NMES on the gastrocnemius and tibialis anterior muscles. Group 2: APAT on lower limbs + NMES on gastrocnemius alone	APAT on the lower limbs	ICU	ICU admission Group 1: 14.10 (6.05) Group 2: 12.85 (4.67) C: 12.20 (4.18)
Batterham et al., 2014 [45]	30	Mixed	I: 42.7 (18–65)^2^ C: 40.5 (19–60)^2^	Two/3 sessions of exercise on a cycle ergometer per week	Standard care	Posthospital discharge	8 wk
Battle et al., 2019 [46]	34	Medical and surgical	I: 61 (49–70)^1^ C: 62.5 (46–70)^1^	Individualized, supervised exercise program with a range of cardiopulmonary, strengthening, and balance exercises	Standard care	Posthospital discharge	6 wk
Berney et al., 2021 [47]	123	Systemic inflammatory response syndrome or sepsis	I: 61 (51–69)^1^ C: 59 (48–67)^1^	Functional electrical stimulation-cycling involved synchronized stimulation of 4 muscle groups 60 min/d for 5 d/wk	Standard care	ICU	ICU admission I: 10 (7–17)^1^ C: 10 (7–17)^1^
Brummel et al., 2014 [48]	87	Medical and surgical	Physiotherapy: 62 (48–67)^a^ Cognitive + physiotherapy = 62 (54–69)^1^ C: 60 (51–69)^1^	Group 1: Once daily physiotherapy session Group 2: Twice-daily 20-min cognitive therapy sessions and once-daily physiotherapy session	Physiotherapy as per hospital protocol	ICU	Hospital admission or until functional independence
Burtin et al., 2009 [49]	58	Medical and surgical	I: 56 (16) C: 57 (17)	Standard care and passive or active exercise training session for on a bedside ergometer 5 d/wk	Standard care: Daily standardized passive or active motion session of upper and lower limbs	ICU	ICU admission I: 11 (5–21)^1^ C: 14 (8–26)^1^
Campos et al., 2022 [50]	47	Surgical	I: 42.4 (14.9) C: 46.7 (17.9)	Early mobilization and NMES	Early mobilization	ICU	ICU admission I: 12 (9–16)^1^ C: 15 (17–21)^1^
Carvalho et al., 2019 [51]	24	Mixed	I: 47.83 (19.61) C: 54.17 (16.71)	Passive exercise sessions with cycle ergometer 20 min once daily	Standard care (standard physiotherapy)	ICU	1 wk
Carvalho et al., 2023 [52]	96	COVID-19	I: 66.63 (14.21) C: 68.31 (12.47)	Functional and respiratory multidisciplinary rehabilitation program. After ICU discharge, the intervention group continued with rehabilitation exercises whether the patient was discharged home or to an inpatient unit	No further rehabilitation intervention after hospital discharge	ICU	12 wk post-ICU discharge
Cebeci et al., 2022 [53]	40	Sepsis	I: 47 (18, 85)^3^ C: 64 (20, 82)^3^	Physiotherapy including passive and active extremity exercises and NMES	Physiotherapy alone	ICU	ICU admission I: 12 (9–16)^1^ C: 15 (17–21)^1^
Chen et al., 2011 [54]	34	Prolonged mechanically ventilated patients	I: 75 (63.0–81.0)^1^ C: 79 (72.5–82.8)^1^	Physiotherapy including diaphragmatic breathing, strengthening exercises, active transfer to a chair, and functional activity training	Standard care	ICU	6 wk supervised, 6 wk unsupervised maintenance program
Chen et al., 2012 [55]	27	Patients in respiratory care	I: 64.9 (21.3) C: 66.5 (18.7)	Exercise training program, including cardiopulmonary endurance training, muscle strengthening, and stretching exercises	Same medical treatment except for exercise training	Post-ICU discharge in hospital	4–6 sessions per week for 10 sessions
Chen et al., 2019 [56]	33	Patients in respiratory care	I: 77.7 (14.3) C:73.8 (17.8)	EMS on the vastus lateralis and rectus femoris of both legs 5 d/wk	Sham stimulation	ICU	2 wk
Chiang et al., 2006 [57]	32	Patients in respiratory care	I: 75 (63.0–80.3)^1^ C: 79 (72.5–82.8)^1^	Physiotherapy including bedside strengthening exercises for the upper and lower extremities and functional activity retraining	Promotion of physical mobilization encouraged verbally	Post-ICU discharge in hospital	6 wk
Connolly et al., 2015 [58]	16	Medical and surgical	I: 66.5 (54.5–73.3)^2^ C: 63.0 (49.5–70.0)^2^	Exercised-based rehabilitation program with a combination of cardiovascular, limb strength, balance, and functional exercises individually tailored for patients: 16 sessions of 40 min, twice weekly	Standard care or observational study	Posthospital discharge	12 wk
Dall’ Acqua et al., 2017 [59]	25	Unknown	I: 56 (13) C: 61 (15)	NMES 30 min session once daily	Sham NMES and conventional physiotherapy	ICU	1 wk or stopped upon extubating
Dantas et al., 2012 [60]	28	Mixed	I: 59.07 (15.22) C: 50.43 (20.45)	The early mobilization group patients received a systematic early mobilisation protocol, twice daily	Passive-assisted and active-assisted exercises according to patient improvement and cooperation	ICU	ICU admission
de Paula et al., 2023 [61]	70	Medical and surgical	I: 61 (48–67)^2^ C: 66 (57–73)^2^	Conventional physiotherapy and an early mobilization protocol. The protocol rates the level of mobilization from 0, indicating no mobilization, to 5, indicating walking with or without assistance	Conventional physiotherapy	ICU	ICU admission I: 5 (4–8.8)^1^ C:5 (4–14.5)^1^
Denehy et al., 2013 [62]	150	Medical and surgical	I: 61.4 (15.9) C: 60.1 (15.8)	Exercise rehabilitation, e.g. resistance movements. Ward/outpatient: cardiovascular, progressive resistance strength training and functional exercise	Mobility included active bed exercises with no outpatient care	ICU	8 wk posthospital discharge
Dong et al., 2014 [63]	55	Mixed	I: 55.3 (16.1) C: 55.5 (16.2)	Rehabilitation therapy daily including progressive activities from heading up actively to walking bedside	Standard care	ICU	Hospital admission or returned to previous level of function or discharged
Eggmann et al., 2018 [64]	115	Mixed	I: 65 (15) C: 63 (15)	Progressive exercise rehabilitation therapy and early mobilization including motor assisted or active cycling, resistance training. Twice or more daily sessions	European standard physiotherapy	ICU	ICU admission
Elliott et al., 2011 [65]	161	Mixed	I: 57.2 (17.0) C: 57.5 (15.1)	Physical rehabilitation program focused on strength training and walking	Standard community-based care after hospital discharge	Posthospital discharge	8 wk
Falavigna et al., 2014 [66]	11	Mixed	I: 34.0 (17.3) C: NA	EMS on the quadriceps muscles and the tibialis anterior, and passive mobilization daily	Passive mobilization of joints on both limbs	ICU	Until the patient attained a force of 4 on the scale of muscle strength for the muscle stimulated
Fischer et al., 2016 [67]	54	Surgical	I: 63.3 (15.5) C: 69.7 (13.1)	NMES on the quadriceps muscle of both thighs twice daily	Sham stimulation	ICU	ICU admission, no longer than 2 wk from postoperative day 1
Fossat et al., 2018 [68]	245	Medical and surgical	I: 65 (13) C: 66 (15)	Standard care and 15-min leg cycling exercise on a cycle ergometer and 50-min EMS of quadriceps muscles delivered each weekday	Standard care: progressive multistep program from passive range of motion exercises to walking	ICU	ICU admission
Gama Lordello et al., 2020 [69]	228	Elective cardiac surgery	I: 57.2 (13.2) C: 58.2 (12.9)	Cycle ergometer for 10 min in addition to arm and leg exercises	Active exercises for lower and upper limbs with each movement in an open kinetic chain	ICU	ICU admission I: 2 (2–3)^2^ C: 2 (2–4)^2^
Gilmartin et al., 2018 [70]	17	Mixed	I: 54.5 (18.88) C: 54.6 (17.34)	Rehabilitation after critical care–assisted discharge pack involving tailored exercise program	Standard physiotherapy care, 1-1 delivered by ward physiotherapist	Posthospital discharge	3 wk starting from ICU discharge
Gruther et al., 2010 [71]	33	Mixed	I: 52 (10) C: 48 (10)	NMES 1 session a day, 5 sessions a week	Sham stimulation	ICU	4 wk
Gruther et al., 2017 [72]	50	Mixed	I: 64 (46–70)^1^ C: 59 (48–70)^1^	Standard care + early rehabilitation including breathing techniques, mobilisation, exercise therapy, and NMES, for 2 h	Standard care: single physiotherapy sessions of mobilization and a mean of 21 min of NMES	Post-ICU discharge in hospital	Hospital admission I: ICU = 23 (12–36)^1^, general ward = 16 (13–23)^1^ C: ICU = 20 (11–33)^1^, general ward = 21 (13–34)^1^
Han et al., 2023 [73]	70	Surgical	I: 66 (57–79)^1^ C: 70 (59–77)^1^	20-min interactive handgrip game twice daily in addition to routinely passive physical rehabilitation	Daily target of 20 min of passive physical rehabilitation	ICU	3 d
Hickmann et al., 2018 [74]	18	Mixed	I: 59 (19) C: 57 (20)	Physiotherapy sessions of continuous passive/active leg chair/bed cycling followed by manual passive/active limbs mobilization	Manual mobilization once a day	ICU	1 wk
Hodgson et al., 2016 [75]	37	Mixed	I: 64 (12) C: 53 (15)	Early goal-directed mobility including active functional activities, comprising rolling, sitting, standing, and walking	All standard unit practice was continued, with no restrictions on physiotherapy or sedation practice	ICU	ICU admission I: 9 (6–17)^1^ C: 11 (8–19)^1^
Jackson et al., 2012 [76]	17	Medical or surgical	I: 47 (41–59)^1^ C: 50 (46–69)^1^	Multicomponent in-home rehabilitation focusing on strength, endurance, and functional ability, included cognitive rehabilitation	No cognitive therapy/speech therapy	Posthospital discharge	12-wk period postdischarge
Jones et al., 2003 [77]	102	Mixed	I: 57 (17) C: 59 (16)	Follow up standard care plus rehabilitation package and a self-directed exercise program	Follow-up call on the general wards post-ICU discharge and ICU follow-up clinic	Posthospital discharge	6 wk
Karatzanos et al., 2012 [78]	52	Mixed	I: 55 (20) C: 59 (21)	EMS was implemented on vastus lateralis, vastus medialis, and peroneus longus of both lower extremities	Patients did not receive EMS	ICU	From the second day after admission until ICU discharge
Kayambu et al., 2015 [79]	42	Sepsis patients	I: 62.5 (30–83)^1^ C: 65.5 (37–85)^1^	Individualized early targeted physical rehabilitation program, 1–2 times daily including EMS, passive/active range of motion	Standard ICU care: physiotherapy strategies provided by the ICU physiotherapist	ICU	ICU admission I: 12.0 (4–45)^1^ C: 8.5 (3–36)^1^
Kho et al., 2015 [80]	29	Medical and surgical	I: 54 (16) C: 56 (18) All: 55 (16)	NMES on 3 muscle groups, physical rehabilitation including progressive mobility exercises	Sham NMES with no electrical stimulation and physical rehabilitation including progressive mobility exercises	ICU	Hospital admission I: 26 (22) C: 35 (20)
Koutsioumpa et al., 2018 [81]	80	Mixed	I: 64 (12.4) C: 66 (13.1)	Transcutaneous NMES and passive or active muscle exercise and respiratory physiotherapy daily	Passive or active muscle exercise and respiratory physiotherapy daily only	ICU	From day 4 to 2 wk in ICU
Kwakman et al., 2022 [82]	33	Medical and surgical	I: 62 (55–68)^1^ C: 64 (54–69)^1^	Standard care and body weight supported treadmill training	Standard care, e.g. ambulation training, active strength exercises, cycling, balance training, and mobilizing out of bed	ICU	Until patient able to ambulate with walking aids and minimal physical support
Leite et al., 2018 [83]	67	Mixed	Diaphragm group: 41.3 (24.26) Quadriceps group: 48.8 (19.69) C: 42.4 (12.74)	NMES therapy in diaphragm or quadriceps group. Received conventional physiotherapy once a day, plus daily session of NMES	Gross motor therapy and respiratory therapy	ICU	ICU admission (ICU diaphragm group: 12.7 (1.92) Quadriceps group: 13.2 (1.87) C: 13.0 (1.64)
Liang et al., 2021 [84]	26	Mixed	All: 62.8	Self-managed music-enhanced exercise intervention that facilitate 5 targeted upper and lower extremities movements	A brochure with instructions to complete the same 5 upper and lower extremity exercises without music	Post-ICU discharge in hospital	Up to 5 d or until hospital discharge from on average 1.2 d post-ICU discharge
Lin et al., 2023 [85]	77	Surgical	I: 53.44 (12.06) C: 52.32 (13.86)	Early goal-directed mobilization	Standard care, e.g. active assistance, active mobilization	ICU	48 h postsurgery
Maca et al., 2023 [86]	39	Mixed	I: 53.0 (16.02) C: 67.0 (12.59)	Standard rehabilitation combined with in-bed cycling exercise, once daily 5 times per week	Standard physiotherapy twice daily	ICU	ICU admission I: 25 (37.93) C: 28 (11.00)
Machado et al., 2017 [87]	38	Mixed	I: 44.64 (19.23) C: 45.13 (18.91)	Conventional therapy + passive exercise on a leg cycle ergometer	Standard care (conventional physical and respiratory therapy)	ICU	I: 12 (7–25)^2^ C: 15 (12–30)^2^
Maffei et al., 2017 [88]	40	Liver transplant	I: 52 (9) C: 54 (9)	Standard care and when awake/extubated, active range of motion and resistance training for the arms and legs was applied	Standard care: passive/active-assistive range of motion exercises for same duration was implemented	ICU	Applied for the duration of stay in the ICU and middle care unit
McCaughey et al., 2019 [89]	20	Mixed	I: 56.5 (18.50) C: 61.0 (17.25)	Abdominal functional EMS	Sham controlled. same as intervention, only difference being the stimulation parameters	ICU	48 h postinitiation of mechanical ventilation to ICU discharge
McDowell et al., 2017 [90]	49	Mixed	I: 51 (13) C: 51 (14)	Personalized exercise program of 2 supervised and 1 unsupervised exercise session per week	No additional support after hospital discharge	Posthospital discharge	6 wk
McWilliams et al., 2016 [91]	63	Mixed	I: 55.0 (12.9) C: 60.8 (12.3)	Outpatient rehabilitation program comprising both exercise and education sessions with physiotherapy team	Physiotherapy, exercises, and education as per current standards of practice until hospital discharge	Posthospital discharge	7 wk
McWilliams et al., 2018 [92]	102	Mixed	I: 62 (46–68)^1^ C: 61 (47–70)^1^	Individualized rehabilitation program with weekly goal setting meetings were held to review progress and update treatment plans as required	Standard care of 30–45 min 5 days a week of physiotherapy until hospital discharge	ICU	One week post-ICU discharge in ward I: ICU = 16 (13–21)^1^, hospital = 29 (22–41)^1^, C: ICU = 18 (12–28)^1^, hospital = 29 (20–46)^1^
Morris et al., 2016 [93]	300	Acute respiratory failure	I: 55 (17) C: 58 (14) All: 56 (15)	Passive range of motion, physiotherapy, and progressive resistance exercises, 3 separate sessions every day of hospitalization	No rehabilitation per treatment protocol	ICU	Hospital admission I: 10.0 (6–17)^1^ C: 10 (7–16)^1^
Moss et al., 2016 [94]	39	Mixed	I: 56 (14) C: 49 (15)	Progressive physiotherapy program including breathing techniques during exercise, progressive range of motion, muscle strengthening, and functional mobility retraining	Upon discharge, only received information on importance of daily exercise	ICU	4 wk
Nakamura et al., 2019 [95]	37	Medical and surgical	I: 76.6 (11.0) C: 74.6 (13.1)	EMS and exercise were introduced throughout the abdomen and all lower extremities between the belts	Maximum possible muscle loading, including range of motion exercise, kicking stability ball, standing exercise and ambulation exercise	ICU	Day 2 to 10
Nakanishi et al., 2020 [96]	36	Medical and surgical	I: 73 (3) C: 66 (3)	EMS on the biceps brachii and rectus femoris muscles 30 min daily	Patients in both groups were mobilized using the same progressive mobilization protocol	ICU	5 d
Nava et al. 1998 [97]	64	COPD recovering from acute respiratory failure	I: 65 (6) C: 67 (9)	Passive mobilization, early deambulation, respiratory and lower skeletal muscle training, complete lower extremity training on a treadmill	Standard medical therapy plus a basic deambulation program	ICU	ICU admission
Nickels et al., 2020 [98]	62	Mixed	I: 56 (18) C: 57 (16)	Standard care and in-bed leg cycling using a cycle ergometer. Passively and progressed to active or resisted exercise	Cycling was not included	ICU	1 wk
Pandey et al., 2013 [99]	127	Mixed	NA	Routine physiotherapy and EMS. EMS was implemented on knee extensors, on tibialis anterior, and of both lower extremities	Routine physiotherapy included the passive movements, active-assisted movements, and chest physiotherapy	ICU	ICU admission
Patel et al., 2023 [100]	198	Medical and surgical	I: 57.9 (42.3–66.8)^1^ C: 54.5 (41.9–64.7)^1^	Early physical and occupational therapy including early mobilization	Physical and occupational therapy delivered when ordered by the primary team	ICU	ICU admission I: 4.7 (3.0–8.9)^1^ C: 5.6 (2.9–9.8)^1^
Patsaki et al., 2017 [101]	128	Mixed	I: 53 (15) C: 53 (16)	NMES was implemented daily on rectus femoris and peroneus longus of both lower extremities	Patients in the control group received sham NMES along with Standard care until hospital discharge	Post-ICU discharge in hospital	Hospital admission I: 22 (22) C: 19 (15)
Pinkaew et al., 2020 [102]	71	Mixed	Group 1: 69.08 (16.96) Group 2: 75.32 (14.28) C: 74.68 (15.23)	Group 1: conventional therapy + early mobilization protocol, progressive passive to active range of motion exercises Group 2: conventional therapy + early mobilization with elastic exercises	Conventional physiotherapy groups included passive and active range of motion exercise, breathing exercise	ICU	ICU admission
Porta et al., 2005 [103]	50	Respiratory intermediate ICU	I: 70 (5.6) C: 72 (5.2)	General physiotherapy and supported arm exercise training using upper arm cycling on the arm ergometer	General physiotherapy	ICU	15 daily sessions
Qie et al., 2023 [104]	190	Mixed	I: 52.52 (3.0) C: 53.78 (2.85)	Progressive early rehabilitation training	Routine nursing, diet guidance and traditional rehabilitation	ICU	ICU admission I: 10.52 (2.28) C: 15.74 (4.24)
Rahiminezhad et al., 2022 [105]	90	Mixed	Group 1: 46.67 (17.24) Group 2: 44.27 (13.61) C: 47.73 (17.63)	Group 1: usual care and passive, active, and active-assistive ROM exercise Group 2: usual care and whole-body massage using the Swedish massage style	Routine physiotherapy	ICU	7 d
Sarfati et al., 2018 [106]	97	Surgical	I: 62 (52–73)^1^ C: 67 (54–75)^1^ All: 64 (53–74)^1^	Standard care + tilting for ≥1 h/d	Standard care: in-bed passive and active range of motion exercises were performed daily in all patients	ICU	ICU admission I: 7 (5–12)^1^ C:10 (6–15)^1^
Schaller et al., 2016 [107]	200	Surgical	I: 66 (48–73)^1^ C: 64 (45–76)^1^ All: 65 (46–74)^1^	Early mobilization no later than 1 day after trial enrolment. Progressive mobilization from passive range of motion to ambulation	Mobilization done in line with the individual centers’ practice guidelines for mobilization and physiotherapy	ICU	ICU admission I: 21 (14–37)^1^ C: 21.0 (14–38)^1^
Schujmann et al., 2020 [108]	99	Mixed	I: 48 (15) C: 55 (12)	Combination of conventional physiotherapy and early and progressive mobilization including exercises for the muscular and cardiorespiratory system	Conventional treatment with active assists and active mobilization as well as bed positioning, bedside, and armchair transfers, orthostatism, and ambulation	ICU	ICU admission I: 5 (4–7)^1^ C: 8 (5–12)^1^
Schweickert et al., 2009 [109]	104	Medical	I: 57.7 (36.3–69.1)^1^ C: 54.4 (46.5–66.4)^1^	Exercise and mobilization via physical and occupational therapy including progressive mobilization starting with passive up to participation in activities of daily living	Standard care with physical and occupational therapy delivered as ordered by the primary care team	ICU	Hospital admission I: 13.5 (8.0–23.1)^1^ C: 12.9 (8.9–19.8)^1^ or until returned to previous level of function
Schweickert et al., 2023 [110]	1917	Medical and surgical	I: 62.0 (16.3) C: 63.0 (16.4)	Mobilization intervention including daily mobilization goals, and interprofessional closed-loop communication	Usual care with no explicit encouragement for daily mobility goals	ICU	ICU admission I: 275.6 (153.4–537.6)^1^ C: 236.5 (140.6–419.6)^1^
Segers et al., 2021 [111]	104	Medical and surgical	All: 60 (15)	Standard care and 1-h NMES session daily	Standard care of physiotherapy and early mobilisation	ICU	1 wk
Shamsi et al., 2019 [112]	36	Mixed	I: 50.66 (11.60) C: 51.83 (7.94)	Transcutaneous NMES for 30 min and stretch 3 times a week	Dorsiflexed the patient’s ankle by applying gentle pressure 3 times a week	ICU	2 wk
Shelly et al., 2017 [113]	28	Medical	I: 59 (50.5–65)^1^ C: 53 (42.75–56)^1^ All: 54.40 (10)	Home-based training with exercise information sheet with respiratory and mobility exercises including active-assisted or active exercises	Medical intervention prescribed by the physician without any formal exercise prescription	Post-ICU discharge in hospital	4 wk
Shen et al., 2017 [114]	25	Medical	I: 77.5 (72–81)^1^ C: 78 (73–83)^1^ All: 78 (72–82)^1^	EMS on both quadriceps and biceps, 32 min/d	Active or passive exercises to individual’s extremities	ICU	Duration of mechanical ventilation I: 6.5 (5–10)^1^ C: 6 (6–15)^1^
Silva et al., 2019 [115]	40	Neurologic	I: 30 (27, 33)^3^ C: 33 (29, 37)^3^	Physiotherapy protocol and NMES bilaterally in the quadriceps femoris, hamstring, tibialis anterior and gastrocnemius muscles was applied once a day for 25 min	Physiotherapy routine protocol including progressive exercises from passive exercises to walking	ICU	2 wk
Veldema et al., 2019 [116]	39	Neurologic	Ergometer training: 62 (13) Resistance training: 58 (9) C: 66 (14)	Ergometer training: Standard care and continuous activity using medical wheelchair ergometer training apparatus Resistance training: interrupted activity at high intensity of resistance exercises	Standard care only	ICU	4 wk
Verceles et al., 2018 [117]	32	Medical	I: 57.1 (12.0) C: 63.1 (11.4)	Multimodal rehabilitation program consisting of progressive, patient specific rehabilitation program of various levels of mobility	Basic rehabilitation activities by physiotherapist	Post-ICU discharge in hospital	8 wk
Waldauf et al., 2021 [118]	88	Mixed	I: 59.9 (15.1) C: 62.3 (15.4)	Progressive mobility program including active exercise daily and functional electrical stimulation or active cycling	Physiotherapy 2 times a day	ICU	ICU admission or 4 wk
Winkelman et al., 2018 [119]	54	Medical	I: 52.68 (18.53) C: 59.48 (15.56)	Early therapeutic mobility twice daily	Early therapeutic mobility once daily	ICU	ICU admission (ETM twice daily = 13.40 (7.97) ETM once daily = 18.76 (14.47)
Wollersheim et al., 2019 [120]	50	Sepsis-related multiple organ dysfunction syndrome	I: 54 (45–68)^1^ C: 45 (39–61)^1^	Protocol-based physiotherapy and NMES and/or whole-body vibration carried out daily	Early in accordance with the physiotherapy protocol	ICU	ICU admission or ≤4 wk in ICU I: 32 (21–48)^1^ C: 26 (17–30)^1^
Wright et al., 2018 [121]	116	Mixed	I: 60 (16) C: 64 (16)	Target delivery of 90 min of physical rehabilitation per day, split between ≥2 sessions	30 min of physical rehabilitation per day	ICU	ICU admission I: 6 (4–9)^1^ C: 5 (4–8)^1^
Wu et al., 2019 [122]	62	Mixed	I: 53.9 (15.0) C: 55.2 (11.4)	Early rehabilitation program individualized for each patient; 2-fold increase in therapy sessions	Standard care, as directed by the acute physicians or surgeons on the acute ward	Post-ICU discharge in hospital	I: 28.0 (14.5–43.5)^1^ C: 14.0 (11.0–14.5)^1^
Yosef-Brauner et al., 2015 [123]	18	Mixed (72% surgical)	I: 51.6 (18) C: 61.5 (12)	Daily progressive exercises of respiratory and functional elements, including muscle-strengthening techniques and maintaining range of motion	Daily custom physiotherapy protocol	ICU	ICU admission I: 13 (4.6) C: 18.11 (3.1)
Yu et al., 2020 [124]	107	Acute respiratory failure	I: 59.98 (8.01) C: 58.37 (7.35)	In-bed cycling combined with passive joint activity, including passive and active exercises	Standard care of turning over every 2 h and releasing both upper limbs for 5 min	ICU	ICU admission I: 11.87 (2.00) C: 13.24 (2.32)
Zanotti et al., 2003 [125]	24	COPD with chronic hypercapnic respiratory failure	I: 66.2 (8) C: 64.5 (4)	Active limb mobilization and EMS 5 d/wk	Active limb mobilization	Post-ICU discharge in hospital	4 wk
Zulbaran-Rojas et al., 2023 [126]	16	COVID-19	I: 66.75 (9.81) C: 62.88 (9.51)	EMS placed on proximal gastrocnemius muscle and Achilles tendon of each leg using a bioelectric stimulation technology microcurrent platform	Nonfunctional EMS acting as placebo	ICU	14 d

Values are mean (SD) unless otherwise specified.

Abbreviations: APAT, active and passive activity training; C, comparator; COPD, chronic obstructive pulmonary disease; EAA, essential amino acids; EMS, electrical muscle stimulation; I, intervention; ICU, intensive care unit; NMES, neuromuscular electrical stimulation; PEPSE, program of enhanced physiotherapy and structured exercise; ROM, Range of Motion

1 Median (IQR).

2 Mean (range).

3 95% CI.

The most common physical rehabilitation intervention was electrical muscle stimulation (EMS) (*n* = 27) [40,41,43,50,53,56,59,66,67,71,[78], [79], [80], [81],^83^,^89^,^95^,^96^,^99^,^101^,^111^,^112^,^114^,^115^,^120^,^125^,^126^], followed by patient mobilization (*n* = 13) [42,60,61,75,85,100,102,[107], [108], [109], [110],^117^,^119^] and cycle ergometry (*n* = 10) [45,49,51,68,69,87,98,103,116,124]. All other interventions can be seen in Table 3. There was considerable variability in the duration of the intervention ranging from 1 d [43] to 3 mo [58].

#### Combined nutritional and physical intervention studies

Six studies investigated combined nutritional and physical interventions, with studies commencing in the ICU (*n* = 4) [[127], [128], [129], [130]] and in the post-ICU period (*n* = 2) [131,132] (Table 4). Interventions included a nutritional supplement combined with enhanced physiotherapy (*n* = 1) [132], increased levels of mobilization, exercise and dietetic therapy (*n* = 1) [131], early mobilization with early nutrition (*n* = 1) [129], and multimodal rehabilitation with increased protein intake (*n* = 1) [128].

**TABLE 4:** Study characteristics of combined nutritional and physical intervention studies.

Citation, year	*n*	Population	Age (y)	Intervention	Comparator	Commencement of intervention	Duration of intervention (d)
de Azevado et al., 2021 [130]	181	Medical and surgical	I: 67.6 (17.8) C: 65.3 (19.7)	Patients undertook 2 daily sessions of cycle ergometry. On the fifth day, protein intake was increased to 2.0–2.2 g/kg/d	On the fifth day, protein intake was increased to 1.4–1.5 g/kg/d and ICU physiotherapy protocol	ICU	Until discharge, death, or 3 wk of stay in the study
Jones et al., 2015 [132]	72	45 y or older	PEPSE + placebo: 64 (13) EAA supplement + no PEPSE: 64 (18) EAA + PEPSE: 62 (14) C: 60 (12)	Glutamine and EAA supplement, early physiotherapy program of enhanced physiotherapy, and structured exercise in addition to self-help program	Placebo nutrition supplement and ICU Recovery Manual	Post-ICU discharge in hospital	12 wk
Kagan et al., 2022 [127]	41	Mixed	Group 1: 63 (18) Group 2: 61 (16) C: 64 (13)	Groups 1 and 2 were randomized to receive EN and protein enriched EN, respectively. Groups 1 and 2 were treated using cycle ergometry	Conventional physiotherapy and EN	ICU	ICU discharge, death, or the completion of 28 d of the study
Verceles et al., 2023 [128]	39	>50 y old	I: 62 (9.3) C: 62 (6.3)	Multimodal rehabilitation plus standard care rehabilitation. Twice per day for 30 min ≤5 d/wk. Muscle strength combined with endurance with concurrent NEMES for 30 min. Protein based on 1.75 g/kg/d	Standard care including PT and OT, energy according to Penn State equation and protein according to ASPEN	ICU	ICU admission or 14 d I: 10.6 (6.3) C: 11.5 (9.2)
Walsh et al., 2015 [131]	228	Mixed	I: 62 (51–71)^1^ C: 62 (53–69)^1^	Higher levels of mobilization, exercise, and relevant dietetic therapy, occupational therapy, and speech and language therapy compared with standard care.	Self-help ICU rehabilitation manual	Post-ICU discharge in hospital	Acute period from ICU discharge through a hospital stay of no longer than 12 wk
Zhou et al., 2022 [129]	150	Mixed	I: Group 1: 57.0 (15.3) I: Group 2: 58.7 (14.9) C: 57.3 (13.7)	Group 1: Early mobilization with early nutritional: Nutrition was initiated within 48 h of ICU admission. Early mobilization was within 24 h of admission Group 2: Early mobilization group: underwent the early mobilization intervention only in addition to standard ICU care	Routine rehabilitation and routine nutritional support	ICU	ICU admission Group 1: 3.4 (3.0–4.9)^1^ Group 2: 4.5 (3.0–7.7)^1^ Control: 4.1 (3.2–6.0)^1^

Values are mean (SD) unless otherwise specified.

Abbreviations: EAA, essential amino acid; EN, enteral nutritional; EMS, electrical muscle stimulation; I, intervention; ICU, intensive care unit; NMES, neuromuscular electrical stimulation; OT, occupational therapy; PT, physiotherapy; PEPSE, program of enhanced physiotherapy and structured exercise; PN, parenteral nutritional.

1 Median (IQR).

#### Nutritional intervention

All (100%) 30 studies of nutritional interventions measured and/or reported ≥1 nutritional variable.

##### Anthropometry (body weight and BMI)

Body weight was measured and reported at baseline in 16 (53.3%) studies [[10], [11], [12], [13],^15^,^19^,^21^,^22^,^25^,^27^,^28^,^31^,^32^,^35^,^36^,^38^]. An additional 12 (40.0%) studies indicated that body weight was measured at baseline, but results were not reported [[16], [17], [18],^20^,^23^,^24^,^29^,^30^,^33^,^34^,^37^,^39^]. Three (9.1%) studies measured and reported body weight at the end of the intervention only [22,35,36]. One study (3%) measured body weight at the end of the intervention and the follow-up period, however did not report results [38]. BMI was measured and reported in 24 (80%) studies at baseline [[10], [11], [12], [13],[15], [16], [17], [18],[21], [22], [23], [24], [25],[28], [29], [30],[32], [33], [34], [35], [36], [37], [38], [39]] and 2 (6.7%) studies at the end of the intervention [35,36].

##### Nutritional screening, nutritional status, and nutritional intake/delivery

Seven (23.3%) studies measured and reported nutritional risk at baseline, whereby 5 studies used the Nutritional Risk Score (NRS)-2002 [12,14,21,25,36] and 2 studies used the Nutritional Risk in Critically ill (NUTRIC) score [21,39], and 1 study classified nutritional risk using BMI categories [23].

Nutritional status was measured and reported in 7 (23.3%) studies at baseline whereby SGA was used in 6 (20%) studies [[16], [17], [18],^21^,^22^,^38^] and 1 used a BMI threshold of <18.5 kg/m^2^, presumed recent weight loss of >10% or physician’s decision to classify malnutrition [28]. One (3.3%) study measured and reported nutritional status at the end of the intervention [22] and another (3.3%) at the follow-up time point [38], both using SGA.

Twenty-eight (93.3%) studies measured and reported energy intake/delivery over the duration of the intervention [[10], [11], [12], [13], [14], [15], [16], [17], [18], [19], [20], [21], [22], [23], [24], [25], [26], [27], [28], [29], [30], [31], [32], [33],^35^,^36^,^38^,^39^], and 4 (13.3%) measured and reported energy intake/delivery during the follow up period [13,28,33,38]. Twenty-one (70.0%) studies measured and reported protein intake/delivery over the duration of intervention [10,11,[13], [14], [15],[17], [18], [19], [20], [21], [22], [23], [24],[26], [27], [28],^33^,^35^,^36^,^38^,^39^], and 3 (10.0%) studies only measured protein intake/delivery during this time [12,16,31]. Four (13.3%) studies measured and reported protein intake/delivery during the follow-up period [13,35,38,133]. Twenty-two (73.3%) studies accounted for these nutritional variables within the outcome analysis [[10], [11], [12], [13], [14], [15], [16], [17],[19], [20], [21], [22], [23],[26], [27], [28], [29],^32^,^33^,^35^,^36^,^39^].

##### Nutritional-related muscle variables

Nutritional-related muscle variables in the nutritional intervention studies were measured in the context of an outcome of the study, as opposed to a measure of nutritional status per se. Five (16.7%) studies measured and reported handgrip strength (HGS) at baseline [13,21,22,38,39], 6 (20%) at the end of intervention [13,14,21,22,38,39], and 6 (20%) at the end of follow-up [13,22,29,33,38,39].

Additional nutritional-related muscle variables were measured and reported in 18 (60%) studies [11,13,16,[21], [22], [23], [24],[26], [27], [28], [29],[33], [34], [35], [36], [37], [38], [39]]. The most common was mid-upper arm circumference in 7 (21.2%) studies [13,16,22,23,29,33,35], followed by muscle thickness in 5 (16.7%) [13,22,34,37,38], muscle volume in 3 (10%) [24,27,28], rectus femoris cross-sectional area in 2 (6.1%) [11,26], fat-free mass in 2 (6.1%) [35,36], muscle strength in 1 (3%) [22], and body composition by bioelectrical impedance in 1 (3%) [36].

#### Physical rehabilitation interventions

##### Outcomes

Overall, 62 (71.3%) physical intervention studies measured and/or reported ≥1 nutritional variable (Table 5) [[40], [41], [42],^44^,^46^,^47^,[49], [50], [51], [52], [53],[56], [57], [58],[60], [61], [62], [63], [64],[66], [69],^71^,^73^,^74^,[78], [79], [80], [81],[83], [84], [85], [86],^88^,^90^,[92], [93], [94], [95], [96],^98^,[100], [103],[105], [111],^114^,^115^,[117], [121],^123^,^125^].

**TABLE 5:** Variables measured and reported in studies of physical, nutritional, and combined interventions promoting physical or functional recovery during and after critical illness.

Variable	All studies, *n* (%)		Nutritional intervention, *n* (%)		Physical intervention, *n* (%)		Combined intervention, *n* (%)	
	Measured	Reported	Measured	Reported	Measured	Reported	Measured	Reported
Body weight	57 (46)	32 (25.8)	28 (93.3)	16 (53.3)	26 (29.6)	16 (18.2)	3 (50.0)	0 (0)
Body mass index	69 (55.7)	63 (50.8)	24 (80)	24 (80)	41 (46.6)	35 (39.8)	4 (66.7)	4 (66.7)
Nutritional screening	10 (8.1)	10 (8.1)	7 (23.3)	7 (23.3)	0 (0)	0 (0)	3 (50.0)	3 (50.0)
Nutritional status	11 (8.9)	9 (7.3)	7 (23.3)	7 (23.3)	2 (2.3)	1 (1.1)	2 (33.3)	1 (16.7)
Energy delivery/intake	41 (33.1)	35 (33.1)	28 (93.3)	25 (83.3)	8 (9.1)	6 (6.8)	5 (83.3)	4 (66.7)
Protein delivery/intake	35 (28.2)	29 (23.4)	25 (83.3)	21 (70)	5 (5.7)	4 (4.6)	5 (83.3)	4 (66.7)
Handgrip strength	40 (32.3)	38 (30.7)	8 (26.7)	8 (26.7)	30 (34.1)	28 (31.8)	2 (33.3)	2 (33.3)
Nutritional-related muscle variables	41 (33.1)	40 (32.3)	18 (60)	18 (60)	21 (23.9)	21 (23.9)	2 (33.3)	1 (16.7)

##### Anthropometry (body weight and BMI)

Bodyweight was measured and reported at baseline in 16 (18.4%) physical intervention studies [47,52,53,56,64,68,71,79,86,88,95,98,103,107,120,125]. An additional 8 (9.2%) studies indicated that body weight was measured at baseline, but results were not reported. Five (5.7%) studies indicated that body weight was measured at the end of the intervention, but none reported the results [41,49,53,69,103].

A total of 35 (42.2%) studies measured and reported BMI at baseline [42,[49], [50], [51], [52], [53],^56^,[61], [62], [63], [64],^68^,^69^,^73^,^74^,[79], [80], [81],^83^,^85^,^86^,^88^,^96^,^98^,^100^,^103^,^106^,^109^,^111^,[117], [118], [119], [120],^125^,^126^], whereas 6 (7.8%) measured BMI without reporting the values [40,41,60,66,71,110]. Three (3.4%) studies measured BMI without reporting the values at the end of the intervention [41,53,69], and 1 (1.1%) study measured BMI at the end of the follow-up period of the intervention [53].

#### Nutritional screening, nutritional status, and nutritional intake/delivery

Only 1 (1.1%) study measured nutritional status at baseline [105]; however, the method was not stated. One study (1.1%) measured and reported both energy and protein intake/delivery over the duration of the intervention [74]. Six (6.9%) studies measured and reported only energy intake/delivery over the duration of the intervention [74,80,81,95,96,109] and 1 (1.1%) study measured only energy intake/delivery over the duration of the intervention [53]. Four (4.6%) studies measured and reported only protein intake/delivery over the duration of the intervention [53,74,95,96]. Four (4.6%) studies accounted for nutritional variables within the outcome analysis [53,74,81,110].

### Nutritional-related muscle variables

The most commonly reported nutritional-related muscle variable was HGS. Notably, this was measured in the context of muscle strength as an outcome of the study, as opposed to a measure of nutritional status per se; 17 (19.5%) studies measured and reported HGS at baseline [46,52,58,67,73,80,84,85,94,101,102,105,114,117,119,120,123] and 26 (29.9%) at the end of the intervention [46,47,52,58,64,67,73,78,80,[84], [85], [86],^90^,^92^,^93^,^98^,^101^,^102^,^105^,^108^,^109^,^114^,^117^,^119^,^121^,^123^]. Two (2.4%) studies measured HGS, without reporting the values at the end of the intervention [49,120] and 12 (13.8%) studies measured and reported HGS at end of the follow-up period [46,47,52,73,80,90,91,93,98,105,117,121].

Additional nutritional-related muscle variables were measure and/or reported in 21 (24.1%) studies [44,47,51,[56], [57], [58],^64^,^66^,^68^,^71^,^74^,^80^,^89^,^96^,^98^,^105^,^111^,^115^,^118^,^120^] and were also included as secondary outcome measures rather than measures of nutritional status (Table 3). Of those that both measured and reported additional nutritional-related muscle variables, muscle thickness was the most common method [8 (9.2%)] [51,56,67,68,71,96,111,115], followed by muscle strength [5 (5.7%)] [57,64,80,105,111] of the tibialis anterior, quadriceps and/or gastrocnemius, and rectus femoris cross-sectional area [3 (3.4%)] [47,58,98], and muscle fiber cross-sectional area [74], skinfold thickness [56], body surface area [120], and fat-free mass and fat-free mass index [58] were measured and reported in 1 (1.1%) study each. One (1.1%) study measured lower leg muscle cross-sectional area [44] but did not report the result.

### Combined nutritional and physical interventions

Five of the 6 studies (83.3%) of combined interventions measured and/or reported ≥1 nutritional variable [[127], [128], [129], [130], [131]].

#### Anthropometry (body weight and BMI)

Three (50.0%) studies indicated that body weight was measured at baseline, but results were not reported [[127], [128], [129]]. BMI was measured and reported at baseline in 3 (50.0%) studies [[127], [128], [129]] and at the end of the intervention in 1 (16.7%) study [130].

#### Nutritional screening, nutritional status, and nutritional intake/delivery

Nutritional risk was measured and reported at baseline in 3 (50%) studies [[128], [129], [130]], with 2 using the NRS-2002 [129,130] and 1 using the NUTRIC score [128]. One (16.7%) study also measured and reported nutritional risk at the end of intervention using the NRS-2002 [130].

Nutritional status was measured at baseline in 2 (33.3%) studies using SGA [129,131], with 1 of these also reporting the SGA at this time point [129]. One (16.7%) study measured and reported nutritional status at the end of intervention, also using SGA [129].

Four (66.7%) studies measured and reported energy and protein intake/delivery over the duration of intervention [127,128,130,131], and a further 1 (16.7%) study measured energy and protein intake over the duration of the intervention but did not report it [129]. Three (50%) studies accounted for nutritional variables within the outcome analysis [129,131,132].

#### Nutritional-related muscle variables

Four (12.1%) studies both measured and reported nutritional-related variables [127,128,130,131]. Two (33.3%) studies measured and reported HGS at the end of intervention [130,131] and 1 (16.7%) at the end of the follow-up period [131]. One (16.7%) study measured and reported thigh muscle volume and cross-sectional area at baseline [128], and another (16.7%) study measured quadricep muscle strength using manual muscle testing at the same time point [127].

### Risk of bias

Only 3studies were considered low risk of bias in all categories. Blinding of participants and personnel was uncommon and had the highest overall risk of bias. Random sequence generation and allocation concealment (selection bias) had the lowest overall risk of bias. Other bias was commonly assessed as unclear (Supplemental Appendix IV**,** Figure 1).

## Discussion

This systematic review aimed to investigate the reporting of nutritional screening, nutritional status, and nutritional intake/delivery in RCTs evaluating nutritional and/or physical rehabilitation interventions to promote physical or physical functional recovery during and following critical illness. Findings suggest that very few studies both measured and reported nutritional variables, specifically nutritional risk (via nutritional screening) and nutritional status, suggesting these measures are not currently considered essential or important components within these trials. Of relevance, the measuring and reporting of all nutritional variables was higher in trials of nutrition interventions than those of physical rehabilitation.

### Nutritional screening and assessment

The prevalence of malnutrition (undernutrition) in the critically ill population ranges from 38% to 78% [134] and is associated with prolonged hospital length of stay and increased morbidity, and mortality [134]. In other populations, malnutrition is also linked to low muscle mass and muscle strength/functional ability and therefore may impact the effectiveness of interventions aiming to improve physical and functional recovery [135]. Indeed, a study investigating the association between body composition and strength and physical function in survivors 1-year following critical illness found that a greater percentage gain in lean mass was associated with increased gait speed and 6-min walking distance [136]. These data indicate that malnutrition and muscle mass are important factors when considering the recovery of physical function in patients who are critically ill.

In clinical practice, risk of developing and diagnosis of malnutrition can be determined using a multitude of individual measures, screening and assessment tools including BMI, Malnutrition Universal Screening Tool, NRS-2002, SGA, and the GLIM criteria [134]. Additionally HGS can be used to independently predict nutritional status and risk of malnutrition [135,137], and measures of fat-free mass via bioelectrical impedance analysis can be used in the GLIM criteria to diagnose malnutrition [138]. However, there is no validated nutritional screening or diagnostic tool for use in critically ill adults, which represents a barrier for use in clinical practice, and likely also in clinical trials, which was reflected in the results of this systematic review.

In this systematic review, the most common nutritional variable that was measured and reported in both nutritional and physical rehabilitation studies was BMI. However, this was only common at baseline rather than other time points. Although considering BMI in these studies may be important as a higher BMI has been associated with an increased likelihood of functional recovery within 6 mo of ICU admission [139], BMI alone does not adequately reflect nutritional status and is unable to recognize patients with obesity (high BMI) who may also have low muscle mass and be at risk of, or have, malnutrition. For this reason, BMI may be best used in the setting as a more comprehensive nutritional screening and assessment tool. Perhaps, unsurprisingly, the measurement and reporting of nutritional screening and nutritional status using available methods other than BMI (e.g. NRS-2002 and SGA) was predominantly undertaken in the studies of nutritional interventions, although the overall number was low. However, nutritional-related muscle variables (e.g. measures of muscle mass and strength) were measured as a primary or secondary outcome and not used as a marker of nutritional status per se.

Besides the lack of a validated tool, several reasons may explain the low use of nutritional screening, nutritional assessment, and nutritional-related muscle variables in trials nutritional interventions and physical rehabilitation. First, accuracy of the variables needed for nutritional screening and assessment (e.g. bodyweight and body composition measures) are questionable in critical illness due to changes in the volume and distribution of total body water [140]. This may lead to an inaccurate classification of patients into the well-nourished category if edema was present. Second, obtaining information such as changes in bodyweight over time, dietary intake, presence of gastrointestinal symptoms and functional capacity (as are required for the SGA and GLIM criteria) is difficult as patients are often sedated and unable to communicate. In particular, body weight is challenging to measure as bed scales are often unavailable, and mobilizing unstable patients to weigh in the critical care environment is difficult. These reasons may also explain why studies performing nutritional assessments were more apparent in the post-ICU population. Nonetheless, given the likely relationship among nutritional status, specifically malnutrition, low muscle mass, and recovery of physical function, measuring and accounting for these factors in clinical trials of nutritional interventions and physical rehabilitation is strongly encouraged.

### Nutritional intake or delivery

Along with nutritional status, nutritional intake/delivery is likely an important factor when considering the physical and functional recovery of ICU survivors. Although currently ICU nutrition guidelines recommend hypocaloric feeding in the acute early phase of critical illness for its potential beneficial effects on clinical outcomes [141], the impact of long-term underfeeding on physical function is unknown. However, it is plausible to suggest that prolonged underfeeding during the period of critical illness may have a negative impact on physical and functional recovery [142] and, therefore, should be measured and reported in studies where physical and functional recovery is the outcome. In this systematic review, most of the nutritional and combined intervention RCTs measured and reported nutritional intake/delivery, whereas this was uncommon in those investigating physical rehabilitation. Where this was reported, energy and protein intake/delivery were suboptimal when compared with nutrition guideline recommendations [74,109]. However, 1 study identified that delivery of higher protein led to a significant improvement in the physical component summary score assessed at 3 and 6 months [130]; acquired weakness also improved, evaluated through HGS at the time of ICU discharge. This may suggest that a higher protein dose, at least when combined with resistance training, may lead to a reduction in muscle wasting. These findings also align with a recent systematic review among critically ill patients who showed a higher protein delivery was associated with muscle loss attenuation by 3.4% per week [143]. Therefore, considering protein intake at a minimum in studies of physical rehabilitation would appear an important component in being able to understand the physical and functional recovery trajectory of ICU survivors.

Finally, it is important to consider that only 1 post-ICU study measured and reported nutritional intake [131]. Further research in this area is a priority as failure to meet nutritional targets post-ICU may negatively impact physical or functional recovery and oral intake in this phase of recovery is known to be poor [144].

### Strengths and limitations

To our knowledge, this is the first systematic review investigating the reporting of nutritional-related variables in nutritional and/or physical interventions in critical illness, which is its major strength. The limitations of this systematic review mainly relate to the quality of the design of the included studies. Specifically, only 3 studies were considered low risk of bias in all categories, with lack of blinding the most prominent source of potential bias. Further, studies investigating interventions of respiratory muscle training were excluded, which may have led to some studies being missed.

### Conclusion

Results of this systematic review found that very few RCTs of physical rehabilitation in the critically ill reported potentially important nutritional variables compared with those investigating nutritional interventions. With an increasing number of patients surviving critical illness, future studies of both nutritional and physical rehabilitation interventions during and following critical illness should measure, report, and account for important nutritional factors that could impact physical and functional recovery.

## Authors’ contributions

The authors’ responsibilities were as follows – RR, DEB, KW, BC, JM: designed this research and formulated the question; RR, DEB, AL, JM: conducted the research; KW: supervised the research; RR: performed data analysis; RR: wrote the paper, which was reviewed by all authors; DEB: had primary responsibility for final content; and all authors: have read and approved the final manuscript.

## Funding

The authors reported no funding received for this study.

## Data availability

Data described in the manuscript, code book, and analytic code will be made available upon request pending application and approval.

## Conflicts of interest

DEB has received consulting fees from Baxter Healthcare and Nutricia. All other authors report no conflicts of interest.
