# Supplementary material for: Reporting of nutritional screening, status, and intake in trials of nutritional and physical rehabilitation following critical illness: a systematic review
Source: Am J Clin Nutr. 2024 Dec 31;121(3):703–23. doi: 10.1016/j.ajcnut.2024.12.028 (PMC11923378; doi:10.1016/j.ajcnut.2024.12.028)
Supplement: multimedia component 1 [file mmc1.docx]

**SUPPLEMENTARY MATERIAL**

**Reporting of nutritional screening, status and intake in trials of nutritional and physical rehabilitation following critical illness: a systematic review**

Reema Rabheru^1,2^, Anne Langan^3^, Judith Merriweather^4,5^, Bronwen Connolly^6,7^, Kevin Whelan^2^, Danielle E. Bear^1,2,8^

^1^Department of Nutrition and Dietetics, Guy’s and St Thomas’ NHS Foundation Trust, London, UK

^2^Department of Nutritional Sciences, King’s College London, London, UK

^3^Department of Nutrition and Dietetics, Barts Health NHS Trust, London, UK

^4^Critical Care, Royal Infirmary of Edinburgh, Edinburgh, UK

^5^Department of Nutritional and Dietetics, Royal Infirmary of Edinburgh, Edinburgh, UK.

^6^Wellcome-Wolfson Institute for Experimental Medicine, School of Medicine, Dentistry and Biomedical Sciences, Queen's University Belfast, Belfast, UK.

^7^Department of Physiotherapy, The University of Melbourne, Australia

^8^Department of Critical Care, Guy’s and St Thomas’ NHS Foundation Trust, London, UK

Corresponding author:

Dr Danielle Bear

Department of Nutritional Sciences, 4^th^ Floor, Franklin Wilkins Building, 150 Stamford Street, London, SE1 9NH, UK

020 7848 3858

[Danielle.e.Bear@kcl.ac.uk](mailto:Danielle.e.Bear@kcl.ac.uk)

### Appendix I: PRISMA Checklist (2020)

| **Section and Topic** | **Item #** | **Checklist item** | **Location where item is reported** |
| --- | --- | --- | --- |
| **TITLE** | | |  |
| Title | 1 | Identify the report as a systematic review. | title |
| **ABSTRACT** | | |  |
| Abstract | 2 | See the PRISMA 2020 for Abstracts checklist. | See checklist |
| **INTRODUCTION** | | |  |
| Rationale | 3 | Describe the rationale for the review in the context of existing knowledge. | 5 |
| Objectives | 4 | Provide an explicit statement of the objective(s) or question(s) the review addresses. | 6 |
| **METHODS** | | |  |
| Eligibility criteria | 5 | Specify the inclusion and exclusion criteria for the review and how studies were grouped for the syntheses. | 7/8 (table 1) |
| Information sources | 6 | Specify all databases, registers, websites, organisations, reference lists and other sources searched or consulted to identify studies. Specify the date when each source was last searched or consulted. | 6/7 |
| Search strategy | 7 | Present the full search strategies for all databases, registers and websites, including any filters and limits used. | supplement |
| Selection process | 8 | Specify the methods used to decide whether a study met the inclusion criteria of the review, including how many reviewers screened each record and each report retrieved, whether they worked independently, and if applicable, details of automation tools used in the process. | 9 |
| Data collection process | 9 | Specify the methods used to collect data from reports, including how many reviewers collected data from each report, whether they worked independently, any processes for obtaining or confirming data from study investigators, and if applicable, details of automation tools used in the process. | 9/10 |
| Data items | 10a | List and define all outcomes for which data were sought. Specify whether all results that were compatible with each outcome domain in each study were sought (e.g. for all measures, time points, analyses), and if not, the methods used to decide which results to collect. | 9/10 |
|  | 10b | List and define all other variables for which data were sought (e.g. participant and intervention characteristics, funding sources). Describe any assumptions made about any missing or unclear information. | 9/10 |
| Study risk of bias assessment | 11 | Specify the methods used to assess risk of bias in the included studies, including details of the tool(s) used, how many reviewers assessed each study and whether they worked independently, and if applicable, details of automation tools used in the process. | 10 |
| Effect measures | 12 | Specify for each outcome the effect measure(s) (e.g. risk ratio, mean difference) used in the synthesis or presentation of results. | n/a |
| Synthesis methods | 13a | Describe the processes used to decide which studies were eligible for each synthesis (e.g. tabulating the study intervention characteristics and comparing against the planned groups for each synthesis (item #5)). | 10 |
|  | 13b | Describe any methods required to prepare the data for presentation or synthesis, such as handling of missing summary statistics, or data conversions. | 10 |
|  | 13c | Describe any methods used to tabulate or visually display results of individual studies and syntheses. | 10 |
|  | 13d | Describe any methods used to synthesize results and provide a rationale for the choice(s). If meta-analysis was performed, describe the model(s), method(s) to identify the presence and extent of statistical heterogeneity, and software package(s) used. | 10 |
|  | 13e | Describe any methods used to explore possible causes of heterogeneity among study results (e.g. subgroup analysis, meta-regression). | 10 |
|  | 13f | Describe any sensitivity analyses conducted to assess robustness of the synthesized results. | n/a |
| Reporting bias assessment | 14 | Describe any methods used to assess risk of bias due to missing results in a synthesis (arising from reporting biases). | n/a |
| Certainty assessment | 15 | Describe any methods used to assess certainty (or confidence) in the body of evidence for an outcome. | n/a |
| **RESULTS** | | |  |
| Study selection | 16a | Describe the results of the search and selection process, from the number of records identified in the search to the number of studies included in the review, ideally using a flow diagram. | 11/12 |
|  | 16b | Cite studies that might appear to meet the inclusion criteria, but which were excluded, and explain why they were excluded. | 12//Figure 1 |
| Study characteristics | 17 | Cite each included study and present its characteristics. | Tables 2, 3,4 |
| Risk of bias in studies | 18 | Present assessments of risk of bias for each included study. | 37 / suppl figure 1 |
| Results of individual studies | 19 | For all outcomes, present, for each study: (a) summary statistics for each group (where appropriate) and (b) an effect estimates and its precision (e.g. confidence/credible interval), ideally using structured tables or plots. | n/a |
| Results of syntheses | 20a | For each synthesis, briefly summarise the characteristics and risk of bias among contributing studies. | 13-37 |
|  | 20b | Present results of all statistical syntheses conducted. If meta-analysis was done, present for each the summary estimate and its precision (e.g., confidence/credible interval) and measures of statistical heterogeneity. If comparing groups, describe the direction of the effect. | n/a |
|  | 20c | Present results of all investigations of possible causes of heterogeneity among study results. | n/a |
|  | 20d | Present results of all sensitivity analyses conducted to assess the robustness of the synthesized results. | n/a |
| Reporting biases | 21 | Present assessments of risk of bias due to missing results (arising from reporting biases) for each synthesis assessed. | n/a |
| Certainty of evidence | 22 | Present assessments of certainty (or confidence) in the body of evidence for each outcome assessed. | n/a |
| **DISCUSSION** | | |  |
| Discussion | 23a | Provide a general interpretation of the results in the context of other evidence. | 37-41 |
|  | 23b | Discuss any limitations of the evidence included in the review. | 41 |
|  | 23c | Discuss any limitations of the review processes used. | 41 |
|  | 23d | Discuss implications of the results for practice, policy, and future research. | 41 |
| **OTHER INFORMATION** | | |  |
| Registration and protocol | 24a | Provide registration information for the review, including register name and registration number, or state that the review was not registered. | Abstract / 6 |
|  | 24b | Indicate where the review protocol can be accessed, or state that a protocol was not prepared. | Abstract / 6 |
|  | 24c | Describe and explain any amendments to information provided at registration or in the protocol. | n/a |
| Support | 25 | Describe sources of financial or non-financial support for the review, and the role of the funders or sponsors in the review. | 42 |
| Competing interests | 26 | Declare any competing interests of review authors. | 42 |
| Availability of data, code and other materials | 27 | Report which of the following are publicly available and where they can be found: template data collection forms; data extracted from included studies; data used for all analyses; analytic code; any other materials used in the review. | n/a |

*From:*  Page MJ, McKenzie JE, Bossuyt PM, Boutron I, Hoffmann TC, Mulrow CD, et al. The PRISMA 2020 statement: an updated guideline for reporting systematic reviews. BMJ 2021;372:n71. doi: 10.1136/bmj.n71. For more information, visit: <http://www.prisma-statement.org/>

### Appendix II: PRISMA Abstract Checklist (2020)

| **Section and Topic** | **Item #** | **Checklist item** | **Reported (Yes/No)** |
| --- | --- | --- | --- |
| **TITLE** | | |  |
| Title | 1 | Identify the report as a systematic review. | Yes |
| **BACKGROUND** | | |  |
| Objectives | 2 | Provide an explicit statement of the main objective(s) or question(s) the review addresses. | Yes |
| **METHODS** | | |  |
| Eligibility criteria | 3 | Specify the inclusion and exclusion criteria for the review. | Yes |
| Information sources | 4 | Specify the information sources (e.g. databases, registers) used to identify studies and the date when each was last searched. | Yes |
| Risk of bias | 5 | Specify the methods used to assess risk of bias in the included studies. | Yes |
| Synthesis of results | 6 | Specify the methods used to present and synthesise results. | Yes |
| **RESULTS** | | |  |
| Included studies | 7 | Give the total number of included studies and participants and summarise relevant characteristics of studies. | Yes |
| Synthesis of results | 8 | Present results for main outcomes, preferably indicating the number of included studies and participants for each. If meta-analysis was done, report the summary estimate and confidence/credible interval. If comparing groups, indicate the direction of the effect (i.e. which group is favoured). | Yes |
| **DISCUSSION** | | |  |
| Limitations of evidence | 9 | Provide a brief summary of the limitations of the evidence included in the review (e.g. study risk of bias, inconsistency and imprecision). | Yes |
| Interpretation | 10 | Provide a general interpretation of the results and important implications. | Yes |
| **OTHER** | | |  |
| Funding | 11 | Specify the primary source of funding for the review. | N/A |
| Registration | 12 | Provide the register name and registration number. | Yes |

*From:*  Page MJ, McKenzie JE, Bossuyt PM, Boutron I, Hoffmann TC, Mulrow CD, et al. The PRISMA 2020 statement: an updated guideline for reporting systematic reviews. BMJ 2021;372:n71. doi: 10.1136/bmj.n71. For more information, visit: <http://www.prisma-statement.org/>

### Appendix III: Online search strategies

Ovid MEDLINE(R) ALL <1946 to March 10, 2022>

1 exp Intensive Care Units/ or exp Intensive Care/ or ICU.mp. or exp Critical Care/ 181876

2 critical illness.mp. or exp Critical Illness/ 40847

3 mechanical ventilation.mp. or exp Respiration, Artificial/ 112582

4 ((intensive or critical) adj1 care unit).mp. [mp=title, abstract, original title, name of substance word, subject heading word, floating sub-heading word, keyword heading word, organism supplementary concept word, protocol supplementary concept word, rare disease supplementary concept word, unique identifier, synonyms] 117548

5 ((intensive or critical) adj1 care).mp. [mp=title, abstract, original title, name of substance word, subject heading word, floating sub-heading word, keyword heading word, organism supplementary concept word, protocol supplementary concept word, rare disease supplementary concept word, unique identifier, synonyms] 248422

6 1 or 2 or 3 or 4 or 5 362774

7 exp Exercise Therapy/ or exercise.mp. or exp Exercise/ 456705

8 ((exercise or physical) adj2 rehabilitation).mp. 9283

9 physiotherapy.mp. 22920

10 physical therapy.mp. [mp=title, abstract, original title, name of substance word, subject heading word, floating sub-heading word, keyword heading word, organism supplementary concept word, protocol supplementary concept word, rare disease supplementary concept word, unique identifier, synonyms] 55659

11 exp Early Ambulation/ or early mobilisation.mp. 3851

12 early mobilization.mp. 3347

13 physical fitness.mp. or exp Physical Fitness/ 39428

14 muscle strength.mp. or exp Muscle Strength/ 56544

15 cycling.mp. 70800

16 electrical muscle stimulation.mp. or exp Electric Stimulation Therapy/ 88363

17 7 or 8 or 9 or 10 or 11 or 12 or 13 or 14 or 15 or 16 713411

18 randomized controlled trial.pt. 560038

19 controlled clinical trial.pt. 94719

20 randomized.ab. 552356

21 randomly.ab. 377078

22 randomised controlled trial.mp. 27985

23 randomised.mp. 119819

24 RCT.mp. [mp=title, abstract, original title, name of substance word, subject heading word, floating sub-heading word, keyword heading word, organism supplementary concept word, protocol supplementary concept word, rare disease supplementary concept word, unique identifier, synonyms] 28973

25 18 or 19 or 20 or 21 or 22 or 23 or 24 1224310

26 6 and 17 and 25 1274

27 exp animals/ not humans.sh. 4966840

28 26 not 27 1236

Ovid MEDLINE(R) ALL <1946 to August 08, 2023>

1 exp Intensive Care Units/ or exp Intensive Care/ or ICU.mp. or exp Critical Care/ 200525

2 critical illness.mp. or exp Critical Illness/ 45653

3 mechanical ventilation.mp. or exp Respiration, Artificial/ 121987

4 ((intensive or critical) adj1 care unit).mp. 134315

5 ((intensive or critical) adj1 care).mp. 276109

6 1 or 2 or 3 or 4 or 5 400574

7 exp Exercise Therapy/ or exercise.mp. or exp Exercise/ 496054

8 ((exercise or physical) adj2 rehabilitation).mp. 10320

9 physiotherapy.mp. 26045

10 physical therapy.mp. 59710

11 exp Early Ambulation/ or early mobilisation.mp. 4024

12 early mobilization.mp. 3696

13 physical fitness.mp. or exp Physical Fitness/ 42392

14 muscle strength.mp. or exp Muscle Strength/ 63305

15 cycling.mp. 80270

16 electrical muscle stimulation.mp. or exp Electric Stimulation Therapy/ 93382

17 7 or 8 or 9 or 10 or 11 or 12 or 13 or 14 or 15 or 16 776416

18 randomized controlled trial.pt. 597601

19 controlled clinical trial.pt. 95398

20 randomized.ab. 612621

21 randomly.ab. 413976

22 randomised controlled trial.mp. 31690

23 randomised.mp. 132086

24 RCT.mp. 34643

25 18 or 19 or 20 or 21 or 22 or 23 or 24 1329106

26 6 and 17 and 25 1437

27 exp animals/ not humans.sh. 5144321

28 26 not 27 1396

29 limit 28 to dt=20220310-20230809 148

### Cochrane search strategy

Search Name: Updated SR 10/03

Last Saved: 10/03/2022 11:56:07

ID Search

#1 "intensive care"

#2 ICU

#3 "critical care"

#4 MeSH descriptor: [Critical Care] explode all trees

#5 "critically ill"

#6 "critical illness"

#7 "mechanical ventilation"

#8 #1 or #2 or #3 or #4 or #5 or #6 or #7

#9 exercise

#10 "physical therapy"

#11 physiotherapy

#12 "early mobilisation"

#13 "physical fitness"

#14 "electrical muscle stimulation"

#15 "functional electrical stimulation"

#16 cycling

#17 hydrotherapy

#18 MeSH descriptor: [Muscle Strength] explode all trees

#19 MeSH descriptor: [Physical Fitness] explode all trees

#20 #9 or #10 or #11 or #12 or #13 or #14 or #15 or #16 or #17 or #18 or #19

#21 "randomised controlled trial"

#22 RCT

#23 "controlled clinical trial"

#24 randomised

#25 randomly

#26 placebo

#27 trial

#28 groups

#29 #21 or #22 or #23 or #24 or #25 or #26 or #27 or #28

#30 #8 and #20 and #29

Search Name: COCHRANE

Date Run: 09/08/2023 00:00:55

ID Search Hits

#1 "intensive care" 40292

#2 ICU 18417

#3 "critical care" 23664

#4 MeSH descriptor: [Critical Care] explode all trees 2676

#5 "critically ill" 8911

#6 "critical illness" 4668

#7 "mechanical ventilation" 13455

#8 #2 or #3 or #4 or #5 or #6 or #7 49438

#9 exercise 129332

#10 "physical therapy" 19238

#11 physiotherapy 21712

#12 "early mobilisation" 1445

#13 "physical fitness" 7387

#14 "functional electrical stimulation" 989

#15 "electrical muscle stimulation" 277

#16 cycling 7225

#17 hydrotherapy 770

#18 MeSH descriptor: [Muscle Strength] explode all trees 8650

#19 MeSH descriptor: [Physical Fitness] explode all trees 4447

#20 #9 or #10 or #11 or #12 or #13 or #14 or #15 or #16 or #17 or #18 or #19 158408

#21 "randomised controlled trial" 651732

#22 RCT 46116

#23 "controlled clinical trial" 184087

#24 randomised 1190137

#25 randomly 314831

#26 placebo 378586

#27 trial 1360606

#28 groups 593871

#29 #21 or #22 or #23 or #24 or #25 or #26 or #27 or #28 1703192

#30 #8 and #20 and #29 2442

### CINAHL search strategy

| S37 | S10 AND S28 AND S36 | 1,463 |
| --- | --- | --- |
| S36 | S29 OR S30 OR S31 OR S32 OR S33 OR S34 OR S35 | Display |
| S35 | TX randomized | Display |
| S34 | TX randomized controlled trial | Display |
| S33 | TX rct | Display |
| S32 | AB randomly | Display |
| S31 | AB randomised | Display |
| S30 | TX controlled clinical trial | Display |
| S29 | TX randomised controlled trial | Display |
| S28 | S11 OR S12 OR S13 OR S14 OR S15 OR S16 OR S17 OR S18 OR S19 OR S20 OR S21 OR S22 OR S23 OR S24 OR S25 OR S26 OR S27 | Display |
| S27 | (MH "exercise") OR (MH "exercise rehabilitation") OR (MH "physical rehabilitation") OR (MH "physical therapy") OR (MH "physiotherapy") OR (MH "mobilisation") OR (MH "early mobilisation") OR (MH "mobilization") OR (MH "early mobilization") OR (MH "physical fitness") OR (MH "electrical muscle stimulation") OR (MH "functional electrical stimulation") OR (MH "cycling") OR (MH "muscle strength") OR (MH "group exercise") OR (MH "hydrotherapy") | Display |
| S26 | TX hydrotherapy | Display |
| S25 | TX group exercise | Display |
| S24 | TX muscle strength | Display |
| S23 | TX cycling | Display |
| S22 | TX functional electrical stimulation | Display |
| S21 | TX electrical muscle stimulation | Display |
| S20 | TX physical fitness | Display |
| S19 | TX early mobilisation | Display |
| S18 | TX mobilisation | Display |
| S17 | TX early mobilization | Display |
| S16 | TX mobilization | Display |
| S15 | TX physiotherapy | Display |
| S14 | TX physical therapy | Display |
| S13 | TX physical rehabilitation | Display |
| S12 | TX exercise rehabilitation | Display |
| S11 | TX exercise | Display |
| S10 | S1 OR S2 OR S3 OR S4 OR S5 OR S6 OR S7 OR S8 OR S9 | Display |
| S9 | (MH "intensive care unit") OR (MH "intensive care") OR (MH "ICU") OR (MH "critical care unit") OR (MH "critical care") OR (MH "critically ill") OR (MH "critical illness") OR (MH "mechanical ventilation") | Display |
| S8 | TX mechanical ventilation | Display |
| S7 | TX critical illness | Display |
| S6 | TX critically ill | Display |
| S5 | TX critical care | Display |
| S4 | TX critical care unit | Display |
| S3 | TX icu | Display |
| S2 | TX intensive care | Display |
| S1 | TX intensive care unit | Display |

CINHAL

S39 S37 AND S38 120

S38 EM 20220311-20230809 462,687

S37 S10 AND S28 AND S36 1,350

S36 S29 OR S30 OR S31 OR S32 OR S33 OR S34 OR S35 423,794

S35 TX randomized 327,699

S34 TX randomized controlled trial 223,496

S33 TX rct 29,956

S32 AB randomly Expanders - Apply equivalent subjects 109,534

S31 AB randomised 45,419

S30 TX controlled clinical trial 11,731

S29 TX randomised controlled trial 29,849

S28 S11 OR S12 OR S13 OR S14 OR S15 OR S16 OR S17 OR S18 OR S19 OR S20 OR S21 OR S22 OR S23 OR S24 OR S25 OR S26 OR S27 421,889

S27 (MH "exercise") OR (MH "exercise rehabilitation") OR (MH "physical rehabilitation") OR (MH "physical therapy") OR (MH "physiotherapy") OR (MH "mobilisation") OR (MH "early mobilisation") OR (MH "mobilization") OR (MH "early mobilization") OR (MH "physical fitness") OR (MH "electrical muscle stimulation") OR (MH "functional electrical stimulation") OR (MH "cycling") OR (MH "muscle strength") OR (MH "group exercise") OR (MH "hydrotherapy") Expanders - Apply equivalent subjects 140,256

S26 TX hydrotherapy 1,966

S25 TX group exercise 2,624

S24 TX muscle strength Expanders - Apply equivalent subjects

Search modes - Boolean/Phrase Interface - EBSCOhost Research Databases

Search Screen - Advanced Search

Database - CINAHL 28,399

S23 TX cycling 14,851

S22 TX functional electrical stimulation 1,283

S21 TX electrical muscle stimulation 159

S20 TX physical fitness 24,333

S19 TX early mobilisation 1,724

S18 TX mobilisation 12,224

S17 TX early mobilization 1,724

S16 TX mobilization 12,224

S15 TX physiotherapy 69,089

S14 TX physical therapy 129,195

S13 TX physical rehabilitation 2,717

S12 TX exercise rehabilitation 644

S11 TX exercise 253,302

S10 S1 OR S2 OR S3 OR S4 OR S5 OR S6 OR S7 OR S8 OR S9 323,395

S9 (MH "intensive care unit") OR (MH "intensive care") OR (MH "ICU") OR (MH "critical care unit") OR (MH "critical care") OR (MH "critically ill") OR (MH "critical illness") OR (MH "mechanical ventilation") Expanders - Apply equivalent subjects 38,107

S8 TX mechanical ventilation 19,941

S7 TX critical illness 19,454

S6 TX critically ill 36,970

S5 TX critical care 197,179

S4 TX critical care unit 4,188

S3 TX icu 45,309

S2 TX intensive care 161,094

S1 TX intensive care unit 112,857

### Embase search strategy

Embase Classic+Embase <1947 to 2022 March 10>

1 critical illness.mp. or exp critical illness/ 40358

2 ICU.mp. or exp intensive care unit/ 312752

3 critical care.mp. or exp intensive care/ 803572

4 mechanical ventilation.mp. or exp artificial ventilation/ 229690

5 ((intensive or critical) adj1 care).mp. [mp=title, abstract, heading word, drug trade name, original title, device manufacturer, drug manufacturer, device trade name, keyword heading word, floating subheading word, candidate term word] 438635

6 ((intensive or critical) adj1 care unit).mp. [mp=title, abstract, heading word, drug trade name, original title, device manufacturer, drug manufacturer, device trade name, keyword heading word, floating subheading word, candidate term word] 278017

7 1 or 2 or 3 or 4 or 5 or 6 1048418

8 exercise.mp. or exp exercise/ 599802

9 ((exercise or physical) adj2 rehabilitation).mp. [mp=title, abstract, heading word, drug trade name, original title, device manufacturer, drug manufacturer, device trade name, keyword heading word, floating subheading word, candidate term word] 9460

10 exp physiotherapy/ or physiotherapy.mp. 119497

11 physical therapy.mp. [mp=title, abstract, heading word, drug trade name, original title, device manufacturer, drug manufacturer, device trade name, keyword heading word, floating subheading word, candidate term word] 37120

12 early mobilisation.mp. or exp mobilization/ 41889

13 early mobilization.mp. 5198

14 physical fitness.mp. or exp fitness/ 47671

15 muscle strength.mp. or exp muscle strength/ 82788

16 exp cycling/ or cycling.mp. 84976

17 electrical muscle stimulation.mp. 479

18 8 or 9 or 10 or 11 or 12 or 13 or 14 or 15 or 16 or 17 885373

19 randomized controlled trial/ 701248

20 controlled clinical trial/ 465488

21 randomized.ab. 802242

22 randomly.ab. 502243

23 randomised controlled trial.mp. 37658

24 randomised.mp. [mp=title, abstract, heading word, drug trade name, original title, device manufacturer, drug manufacturer, device trade name, keyword heading word, floating subheading word, candidate term word] 173968

25 RCT.mp. [mp=title, abstract, heading word, drug trade name, original title, device manufacturer, drug manufacturer, device trade name, keyword heading word, floating subheading word, candidate term word] 48376

26 19 or 20 or 21 or 22 or 23 or 24 or 25 1709705

27 7 and 18 and 26 5057

Embase Classic+Embase <1947 to 2023 August 09>

1 critical illness.mp. or exp critical illness/ 44168

2 ICU.mp. or exp intensive care unit/ 369135

3 critical care.mp. or exp intensive care/ 896481

4 mechanical ventilation.mp. or exp artificial ventilation/ 267949

5 ((intensive or critical) adj1 care).mp. [mp=title, abstract, heading word, drug trade name, original title, device manufacturer, drug manufacturer, device trade name, keyword heading word, floating subheading word, candidate term word] 499815

6 ((intensive or critical) adj1 care unit).mp. [mp=title, abstract, heading word, drug trade name, original title, device manufacturer, drug manufacturer, device trade name, keyword heading word, floating subheading word, candidate term word] 327184

7 1 or 2 or 3 or 4 or 5 or 6 1180120

8 exercise.mp. or exp exercise/ 659412

9 ((exercise or physical) adj2 rehabilitation).mp. [mp=title, abstract, heading word, drug trade name, original title, device manufacturer, drug manufacturer, device trade name, keyword heading word, floating subheading word, candidate term word] 10695

10 exp physiotherapy/ or physiotherapy.mp. 132454

11 physical therapy.mp. [mp=title, abstract, heading word, drug trade name, original title, device manufacturer, drug manufacturer, device trade name, keyword heading word, floating subheading word, candidate term word] 41565

12 early mobilisation.mp. or exp mobilization/ 45413

13 early mobilization.mp. 5701

14 physical fitness.mp. or exp fitness/ 51276

15 muscle strength.mp. or exp muscle strength/ 94510

16 exp cycling/ or cycling.mp. 95508

17 electrical muscle stimulation.mp. 543

18 8 or 9 or 10 or 11 or 12 or 13 or 14 or 15 or 16 or 17 975836

19 randomized controlled trial/ 780104

20 controlled clinical trial/ 471098

21 randomized.ab. 891568

22 randomly.ab. 552823

23 randomised controlled trial.mp. 42644

24 randomised.mp. [mp=title, abstract, heading word, drug trade name, original title, device manufacturer, drug manufacturer, device trade name, keyword heading word, floating subheading word, candidate term word] 192807

25 RCT.mp. [mp=title, abstract, heading word, drug trade name, original title, device manufacturer, drug manufacturer, device trade name, keyword heading word, floating subheading word, candidate term word] 56990

26 19 or 20 or 21 or 22 or 23 or 24 or 25 1871121

27 7 and 18 and 26 5898

28 limit 27 to dc=20220311-20230809 941

### Web of Science search strategy

<https://www.webofscience.com/wos/woscc/summary/e12eee2d-5530-4ef3-b840-d77da30df0aa-270160c4/relevance/1>


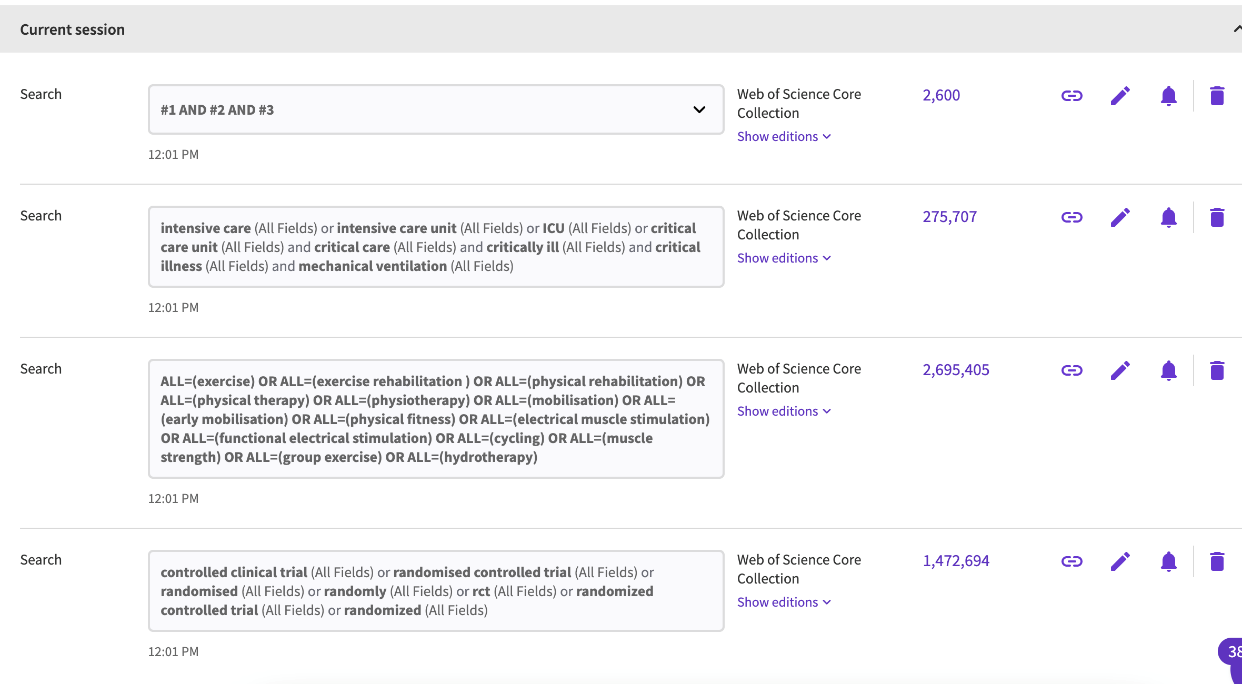


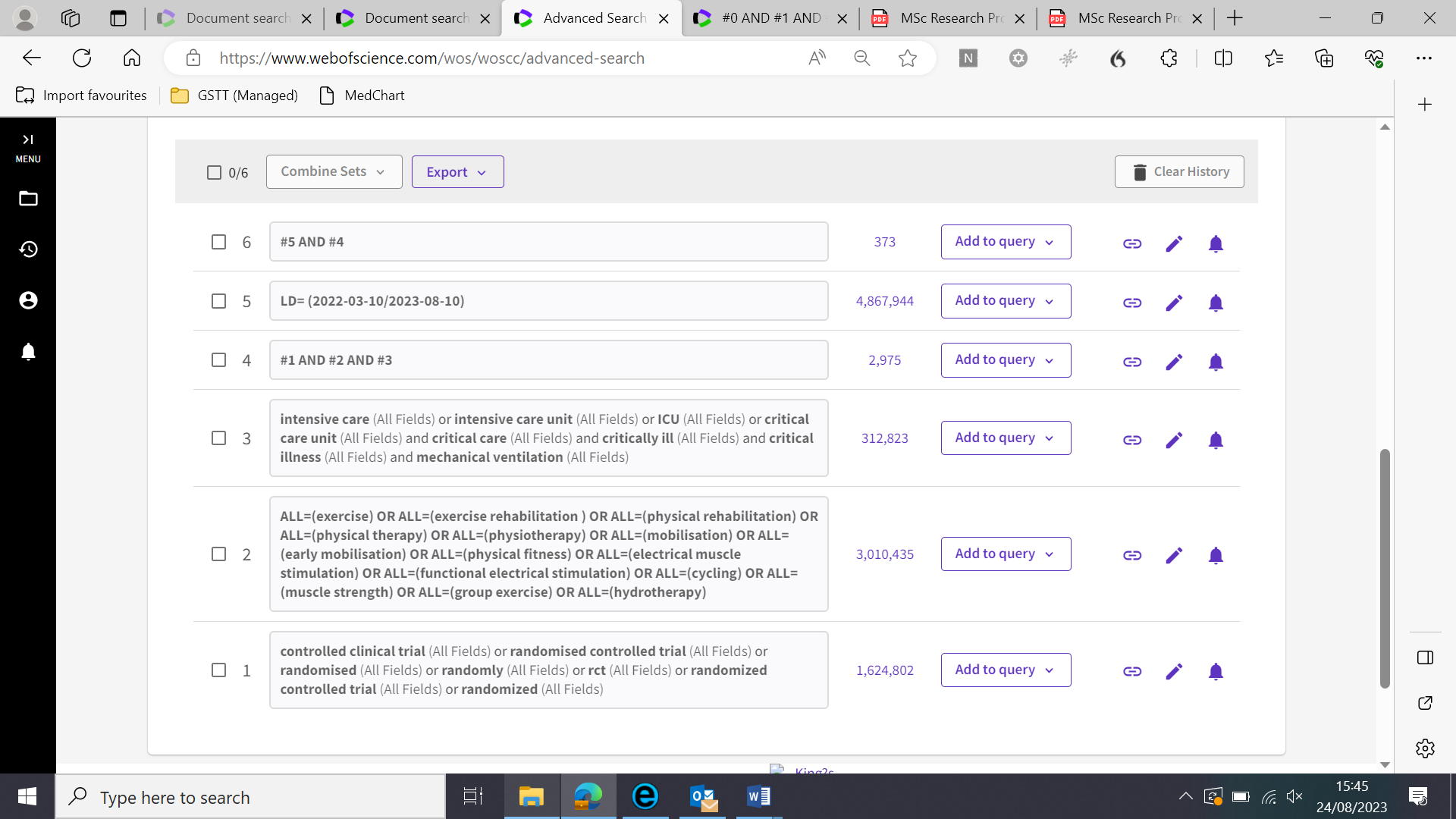


<https://www.webofscience.com/wos/woscc/summary/c26957f2-33b7-4097-9ddc-c0040aa8b512-9f8d3aab/relevance/1>

**APPENDIX IV: Risk of Bias Summary for all Studies and Outcomes**


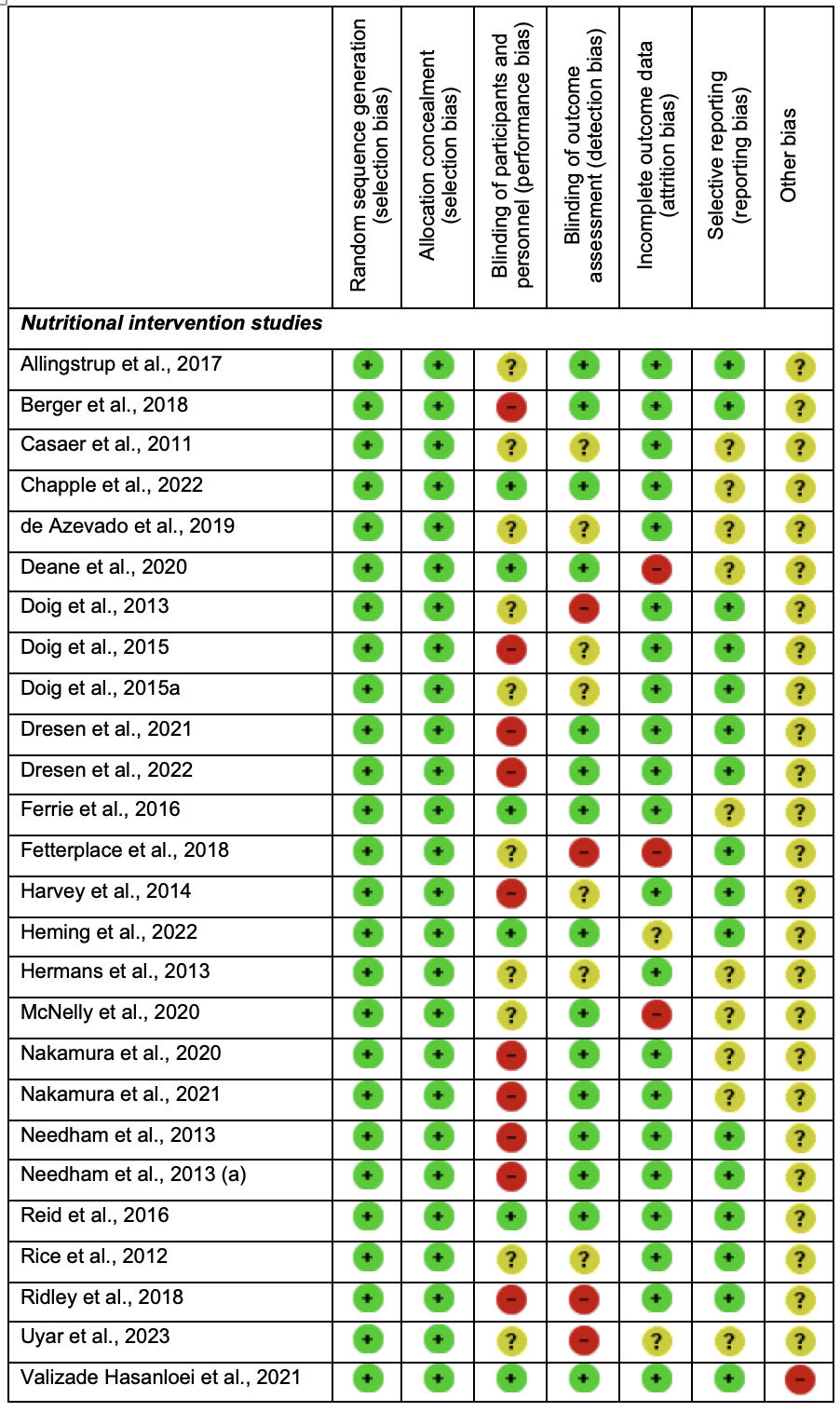


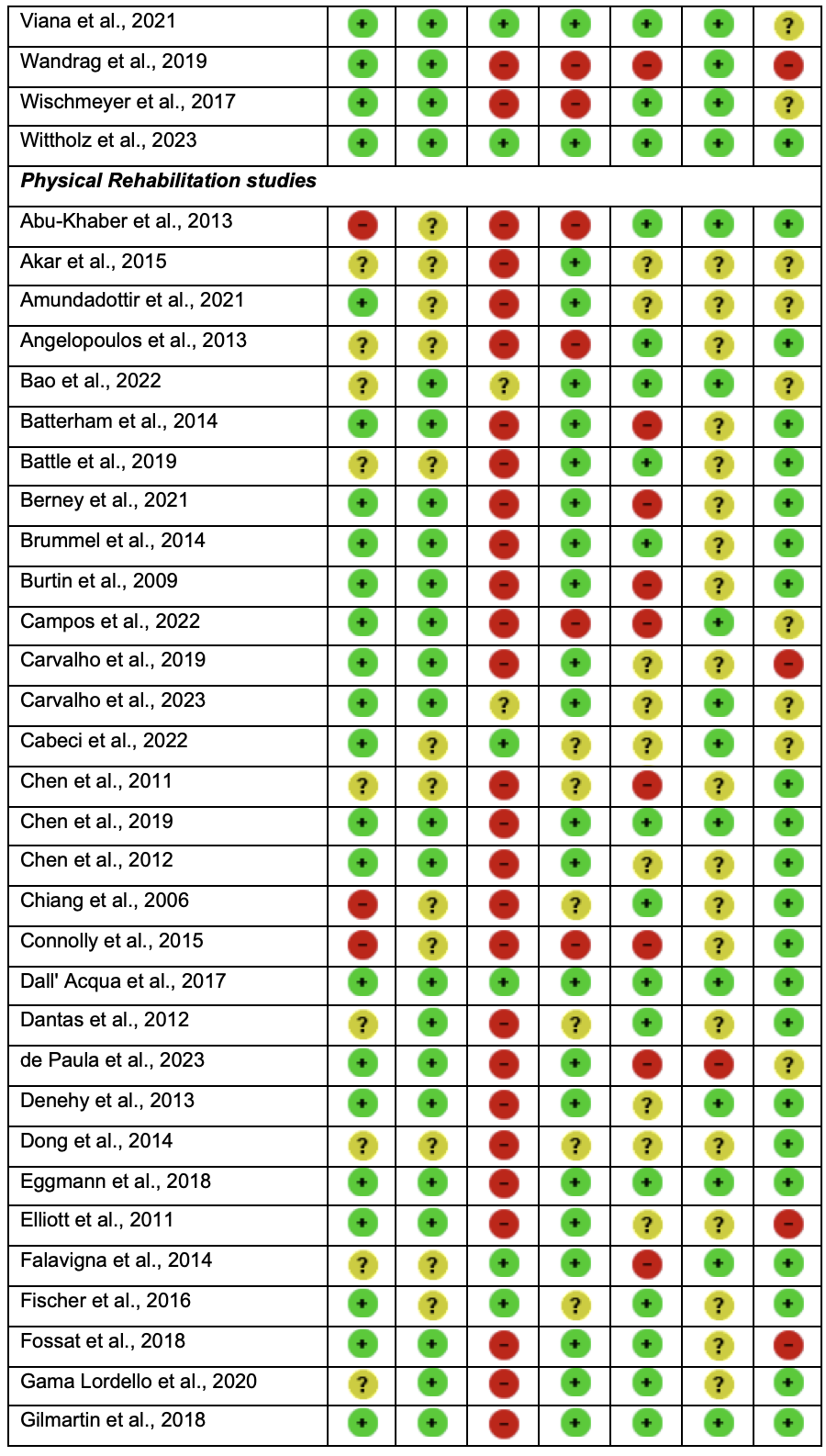


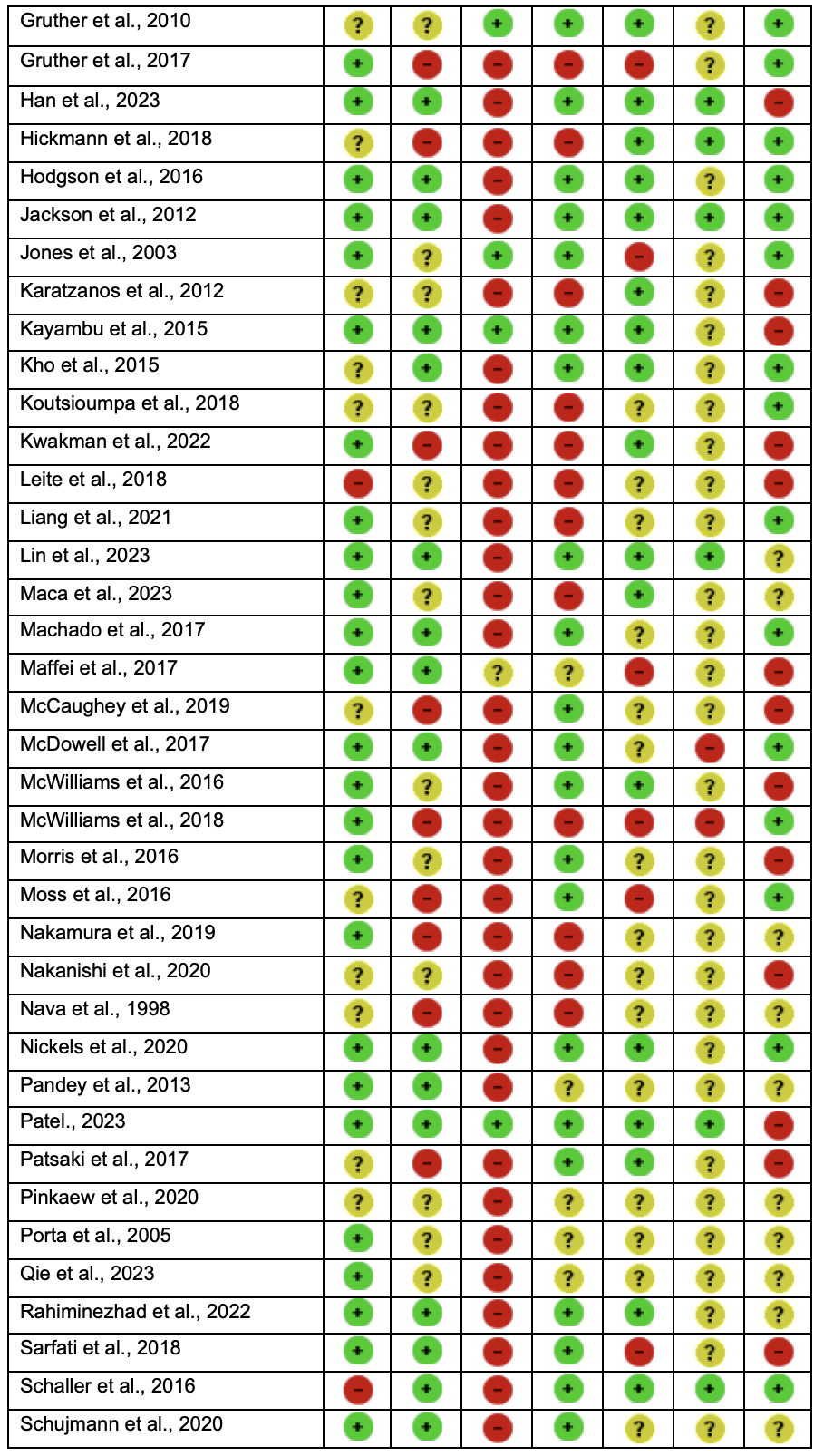


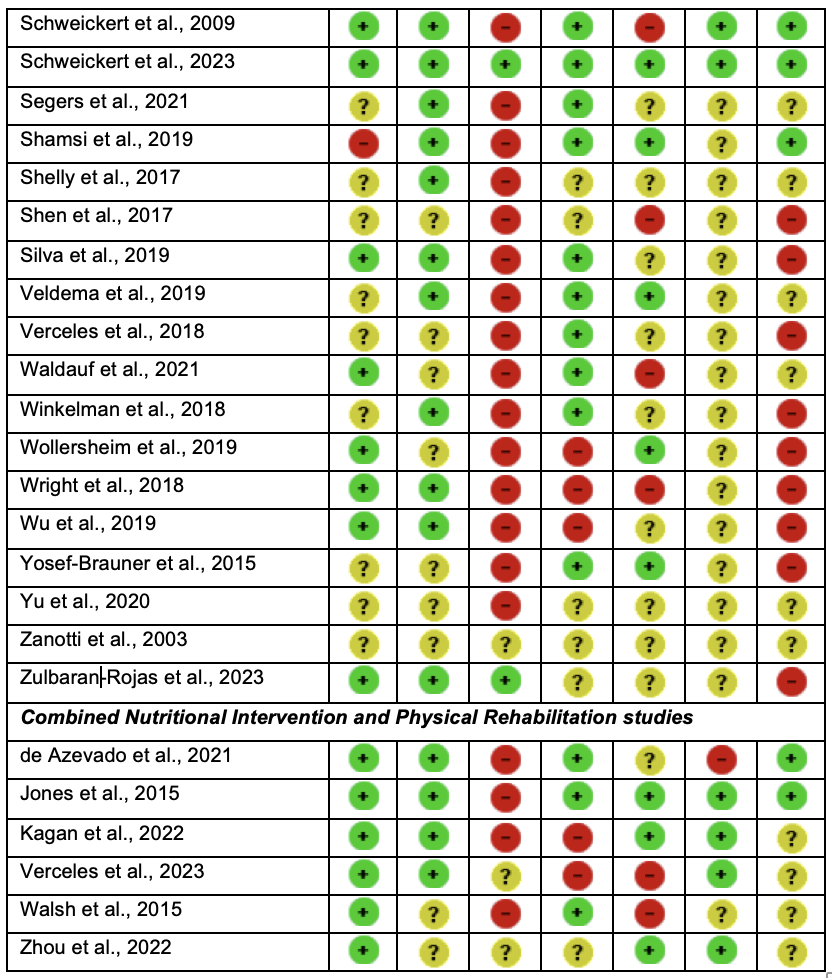


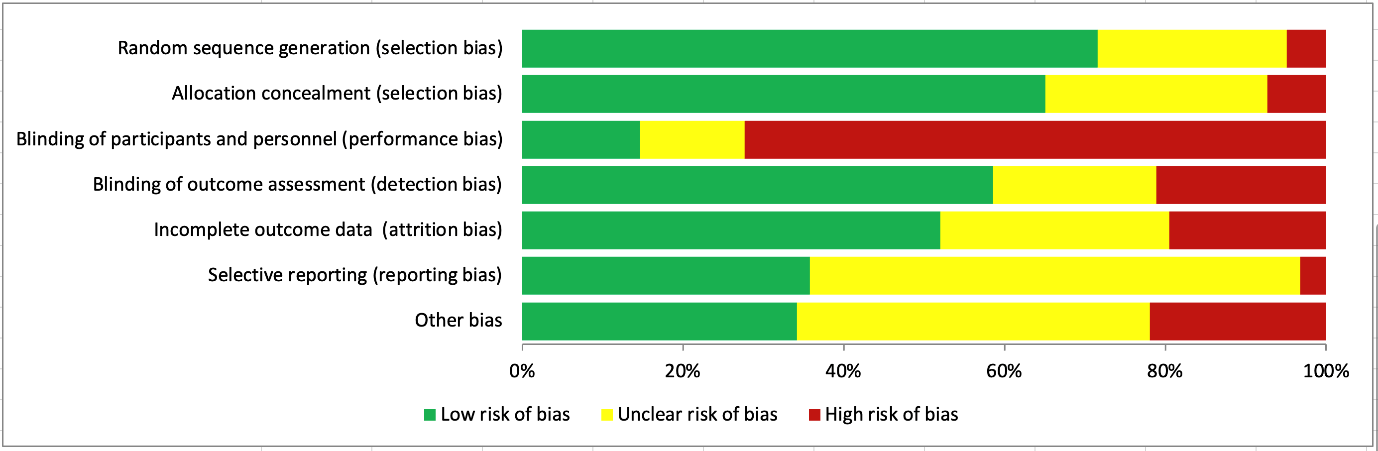


**Supplementary Figure 1: Risk-of-bias summary for all studies and outcomes**.
